# Challenges and Opportunities of Targeting Astrocytes to Halt Neurodegenerative Disorders

**DOI:** 10.3390/cells10082019

**Published:** 2021-08-07

**Authors:** Chiara F. Valori, Agostino Possenti, Liliana Brambilla, Daniela Rossi

**Affiliations:** 1 Molecular Neuropathology of Neurodegenerative Diseases, German Centre for Neurodegenerative Diseases (DZNE), 72076 Tübingen, Germany; 2 Laboratory for Research on Neurodegenerative Disorders, Istituti Clinici Scientifici Maugeri IRCCS, 27100 Pavia, Italy; agostino.possenti@icsmaugeri.it (A.P.); liliana.brambilla@icsmaugeri.it (L.B.)

**Keywords:** neurodegenerative diseases, astrocytes, neuroinflammation, rehabilitation, targeted therapy

## Abstract

Neurodegenerative diseases are a heterogeneous group of disorders whose incidence is likely to duplicate in the next 30 years along with the progressive aging of the western population. Non-cell-specific therapeutics or therapeutics designed to tackle aberrant pathways within neurons failed to slow down or halt neurodegeneration. Yet, in the last few years, our knowledge of the importance of glial cells to maintain the central nervous system homeostasis in health conditions has increased exponentially, along with our awareness of their fundamental and multifaced role in pathological conditions. Among glial cells, astrocytes emerge as promising therapeutic targets in various neurodegenerative disorders. In this review, we present the latest evidence showing the astonishing level of specialization that astrocytes display to fulfill the demands of their neuronal partners as well as their plasticity upon injury. Then, we discuss the controversies that fuel the current debate on these cells. We tackle evidence of a potential beneficial effect of cell therapy, achieved by transplanting astrocytes or their precursors. Afterwards, we introduce the different strategies proposed to modulate astrocyte functions in neurodegeneration, ranging from lifestyle changes to environmental cues. Finally, we discuss the challenges and the recent advancements to develop astrocyte-specific delivery systems.

## 1. Introduction

Neurodegenerative conditions are often fatal, untreatable neurological disorders greatly impairing the quality of life of the affected patients and their caregivers. With the aging of the western population, epidemiologists estimate that the cumulative prevalence of these diseases will triplicate by the year 2050 [1] (https://www.prb.org/global-dementia/ https://www.prb.org/resources/dementia-cases-expected-to-triple-by-2050-as-world-population-ages/, accessed on 5 August 2021). Establishing effective therapy protocols to slow down or halt neurodegeneration is therefore a need that requires urgent attention. In the past, the strategies exploited to achieve this goal were mainly based on the administration of non-cell-specific drugs or drugs acting on pathogenic mechanisms taking place within neurons. Neuron-centric approaches stem from an early vision according to which insults underlying the neurodegenerative process occur only inside neurons, causing their demise. In this paradigm, surrounding glial cells are merely adapting by acquiring a reactive phenotype, a phenomenon commonly known as “reactive gliosis”. Growing evidence emphasizes the limits of this concept and suggests a more active role of glial cells in the central nervous system (CNS) functioning. Preliminarily, our increased understanding of the CNS activity reveals the existence of a great variety of glial cells and highlights the exquisite complexity of their interactions with neurons. Among glia, astrocytes emerge as a critical and highly heterogeneous population that perform a wide array of functions, spanning from maintaining water and ion homeostasis to modulating neuronal transmission (reviewed in [2,3]). In addition to these fundamental interactions with neurons, they exchange signals also with other glial cell populations and they intimately interact with the blood capillaries to maintain the blood-brain barrier (BBB, [4]) and the glymphatic system [5]. Considering the importance of astrocyte functions for CNS performance, it is reasonable to postulate that astrocyte dysfunction can be a primary event of a pathogenic cascade ultimately leading to neuronal loss. An example that corroborates this view is the case of Alexander’s disease [6], a lethal leukodystrophy caused by mutations in the glial fibrillary acidic protein (*GFAP*) gene, which encodes an astrocytic intermediate filament protein. In keeping with this, autoantibodies against the astrocytic water channel protein Aquaporin-4 (AQP4) were shown to cause ~80% of occurrences of neuromyelitis optica, an autoimmune condition with a neurodegenerative component [7]. Finally, recent findings suggest that neurotrophic viruses, such as Zika and West Nile viruses, but also SARS-CoV-2, harm the CNS by interfering with astrocyte functions [8]. This amount of evidence provides a first argument challenging the neuron-centric dogma. The second element of confutation of the neuron-centric hypothesis is represented by the evidence that astrocyte dysfunction can also play a key role in the pathogenesis of several chronic neurodegenerative conditions, including Alzheimer’s (AD, [9]), Parkinson’s (PD, [10]), Huntington’s diseases (HD, [11]), and Amyotrophic Lateral Sclerosis (ALS, [12]). A third indication supporting the importance of glial cells in the diseased CNS is based on the growing awareness that “reactive gliosis” cannot be simply considered a stereotyped passive “reaction” to an insult. Rather, it is a highly articulated process where microglia, peripheral immune cells, and astrocytes sense neuronal distress and exchange signals to mount a tailor-made inflammatory milieu in response to distinct forms of injuries. This phenomenon gives rise to a neuroinflammatory reaction that can have either a neuroprotective or a detrimental impact on neurons, depending on the nature and the persistency of the insult (reviewed in [13]). 

This evidence suggests that glial cells must be included in any working model exploited to investigate pathological conditions of the CNS, and should guide the development of innovative therapies to hinder neurodegeneration. Among glial cells, astrocytes appear to be a particularly appealing target [14]. In this review, we first introduce the many aspects of astrocyte biology providing a rationale to investigate their therapeutic potential. Then, we discuss how targeting astrocytes proved successful in preclinical models of different neurodegenerative diseases. Since a weapon is only as good as its targeting system is, we present the recent advancements to develop cell-specific delivery of therapeutics. Finally, we introduce proof-of-principle trans-differentiation studies demonstrating that astrocytes can become neurons and potentially restore lost functions.

## 2. Many Astrocytes, Many Functions in Health

The first description of astrocytes dates back to the XIX century when neuropathologists reported their morphological heterogeneity in different CNS areas. However, it is only in recent years that we started to understand the implications of these early observations. Developmental studies clarified that astrocytes differentiate from radial glia in the ventricular zone, migrate radially, but not laterally, to colonize distinct domains in a process known as “tiling”. At these sites, they integrate signals from the surrounding neurons to reach a maturation status apt at supporting the specific type of neurons they interact with [15,16,17,18,19,20,21]. More recently, the implementation of transcriptome sequencing technologies at a single-cell resolution (scRNAseq) and in situ hybridization provided indisputable evidence that astrocytes display distinct gene expression profiling not only in different CNS regions, but even within the same area [22,23,24,25,26,27,28]. This diversification may depend also on the gender of the animals taken under consideration [29,30]. In keeping with this, functional studies demonstrated that astrocytes display distinct area- and gender-dependent functional performances [27,31,32,33,34,35]. In humans, astrocyte variety can be even more pronounced than in rodents, considering that their morphology [36] and transcriptional signatures [37,38,39] are remarkably more complex.

Taken together, this amount of evidence is full of implications. Firstly, it suggests that we should refine the definition of “astrocytic marker” by critically assessing whether the alleged marker would indeed label all astrocytes or one or more subpopulations. This implies that pathologists and molecular biologists should reach a consensus to identify markers of astrocyte-subpopulations, which would help to attribute individual functions to each type. In fact, we cannot, *a priori*, assume that every astrocyte would perform all the “astrocytic” functions. Secondly, astrocyte identity is species-specific and determined not only by the unfolding of an intrinsic developmental program but also by distinct cues they receive from the surrounding cells. This implicates that we need to proceed with caution when evaluating the phenotype of astrocytes in monoculture. Furthermore, it supports the requirement to validate in human material any finding obtained in animal models. Related to this latter point, it is important to mention that the use of astrocytes differentiated from human inducible pluripotent stem cells (hiPSCs) is a rapidly expanding approach that has come in handy to investigate their role in physiological and pathological contexts (reviewed in [40]). Remarkably, astrocytes differentiated from hiPSCs by different protocols display regional-specific differential gene expression [41] and retain the aging features of the original host [42]. Furthermore, hiPSCs can generate 3D cultures, known as organoids, mimicking the development of different brain areas and allowing the study of cell-cell interactions (reviewed in [43]). Intriguingly, a recent scRNAseq study analyzing ~20.000 cells from cerebral organoids identified 4 distinct astrocyte subpopulations [44]. Yet, several aspects of the astrocyte biology in the context of organoid maturation still deserve in-depth investigations. For instance, issues that remain to be clarified include: (i) how organoid-astrocytes are molecularly and functionally close to CNS-astrocytes and (ii) whether astrocytes isolated from specific organoids mimic those from the corresponding brain regions.

## 3. Many Astrocytes, Many Functions in Disease 

Upon injury and disease of the CNS, astrocytes, and microglia become “reactive”, i.e., they undergo several morphological, transcriptional, biochemical, metabolic, and functional changes. This response, commonly referred to as “reactive gliosis”, is characterized by the generation of an inflammatory environment into the nervous tissue. The nature and the role of such neuroinflammatory reaction is a timely and highly debated issue, which has been discussed to great extent elsewhere (reviews [13,45,46,47,48]). Here, we will specifically focus on the features and roles of reactive astrocytosis in order to unveil whether and how modulating this process can be beneficial in various pathological conditions. There are a few key concepts that should be carefully considered when studying this phenomenon. Firstly, reactive astrocytosis is a highly specialized response driven by the type, the persistency, and the context of the insult. Secondly, it can be modulated by many environmental cues coming from within and outside the CNS. 

Several studies have been undertaken to standardize the classification of reactive astrocytes. In one early investigation, Zamanian et al. analyzed the transcriptome of astrocytes subject either to a neuroinflammatory or to an ischemic insult, thereby identifying two distinct molecular signatures providing a neurotoxic (A1) or a neuroprotective (A2) phenotype, respectively [49]. Interestingly, activated microglia were reported to trigger the A1 phenotype by releasing cytokines, such as IL-1α, TNFα, and C1q [32,50] and by shedding mitochondrial fragments [51]. Furthermore, microglial expression of the membrane molecules Semaphorin 4D and Ephrin-B3 was described to control the astrocyte responses via the interaction with the respective Plexin B1/B2 and EphB3 receptors on the astrocytes [52]. Microglia were also shown to regulate astrocytic phagocytosis of neuronal debris [53]. Although A1 astrocytes were initially identified in aging and several chronic neurodegenerative conditions [50,54,55,56,57]), other studies failed to corroborate these findings [58,59,60,61,62,63,64]). These discrepancies led to the question of whether the A1 phenotype could be universally implemented to define the neurotoxic status of astrocytes [65]. Also, the A1/A2 dichotomy was challenged, and it was proposed that this classification may pinpoint the extreme ends of a continuous phenotypic spectrum [66,67]. In keeping with this, astrocytes were reported to display a Gaussian distribution when classified on the basis of their inflammatory status in a mouse model of experimental autoimmune encephalomyelitis (EAE) [52]. This led to the formulation of an alternative conceptual frame to guide the investigations on astrocyte reactivity [13]. Firstly, it was proposed that experiments should be designed and interpreted taking many variables into consideration, i.e., the intrinsic diversity of astrocyte subpopulations, their proliferation rate, the type, and the persistence of the injury as well as coupling “omic” data with morphological analysis. Secondly, astrocytosis was suggested to account for a collection of alternative states transiently altering the transcriptome of the astrocyte subpopulations. Finally, any correlative evidence was intended to require experimental validation [13]. Following this path, Yu et al. undertook a systematic investigation of the transcriptional changes induced in mouse striatal astrocytes by an array of 14 different insults, thereby demonstrating that astrocytes mount tailor-made responses to each specific injury [64]. Besides, astrocytes display sexual dimorphisms in their responses to pathological stimuli and neuroinflammation [34,68,69], thus likely contributing to sex-dependent differences in the penetrance and progression of neurodegenerative conditions and aging [70]. Recent findings unveiled a fascinating connection also between inflammation and regulation of circadian rhythms. In particular, the transcription factor Bmal1 was identified as a crucial element in both the maintenance of circadian rhythms and reactive astrocyte-driven inflammation [71]. It is therefore unsurprising that pharmacological treatments with the steroid hormone dehydroepiandrosterone (DHEA), which acts on Bmal1, may play a strong anti-inflammatory activity on astrocytes and microglia [72] as well as alter the circadian rhythms of these cells [73]. Furthermore, Bmal1 depletion from astrocytes leads to motor and cognitive alterations and shortens the lifespan in transgenic mice [74,75]. Considering that circadian arrhythmias can contribute to the development of neurological conditions and recognize symptoms of different neurodegenerative diseases [76], we speculate that astrocytes might be the primary source of impairment. Further investigations are, however, required to dissect the contribution of inflammation to circadian rhythm alteration and, therefore, to develop a suitable therapeutic intervention.

Besides reactive astrocytosis, there are other aspects of astrocyte pathology that deserve attentive consideration. It has been recently proposed that astrocyte loss-of-function is the main feature of glial dysfunction in the aging brain (reviewed in [65]). In addition, neuropathologists are identifying atrophic and even degenerating astrocytes in an ever-growing list of neurological conditions, including Aicardi-Goutieres syndrome [77], AD [78,79], PD [80], epilepsy [81], spinal cord injury [82], brain ischemia [83], and ALS [84,85,86]. Importantly, pharmacological treatments preventing astrocyte degeneration were reported to successfully ameliorate the phenotype of a mouse model of ALS [84,85], thus suggesting that the loss of astrocytes contributes to the pathogenesis of this disease.

On the basis of all these studies, we can conclude that the astrocyte response to neurodegeneration consists of both astrocyte loss- and gain-of-functions. Furthermore, we can now speculate that such diverse reactions may represent the distinct alterations of different astrocyte subpopulations.

## 4. Astrocyte Replacement Therapy 

**Figure 1:**
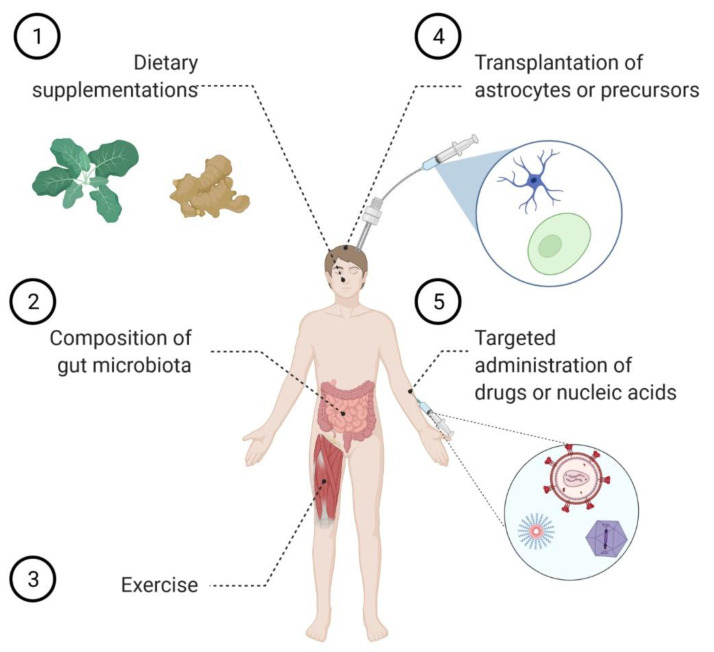
The many ways to reach the stars. Several possible strategies can be endorsed to boost the protective activities of the astrocytes towards neurons. These include changing the lifestyle by (**1**) including in the diet foods rich in antioxidants, such as broccoli and turmeric, (**2**) controlling the gut microbiota, and (**3**) regularly performing physical exercise. In the case of neurodegenerative conditions, i.e., when astrocytes themselves undergo degeneration, (**4**) transplantation of either astrocytes or their precursors can provide therapeutic benefits. Alternatively, (**5**) aberrant pathways can be precisely targeted by using advanced delivery systems, such as functionalized nanoparticles, homing peptides, or viruses, to convey drugs or nucleic acids into the astrocytes in order to correct the altered signaling cascades. (Created with BioRender.com).

Several lines of evidence indicate that astrocytes display a powerful homeostatic activity under physiological conditions, while they show several dysfunctions in pathology. Thus, it was hypothesized that cell replacement therapy may act as a valuable therapeutic approach to tackle neurodegenerative conditions (Figure 1). As prerequisites for a successful clinical implementation of this strategy, preclinical studies were performed and demonstrated that not only healthy astrocytes can be implanted into the diseased CNS, but they can resist the neuroinflammatory environment in which they become embedded. Furthermore, different studies reported the beneficial impact on disease progression and survival of transplanting either astrocytes in rodent models of AD [87] and PD [88,89,90], or healthy glial cell precursors in models of ALS [91,92,93], dementia with Lewy bodies [94], and subcortical white matter stroke [95]. These encouraging results prompted the registration of a few phase I/IIa clinical trials to investigate the safety and efficacy of different transplantation strategies. In particular, ALS patients received an intrathecal administration of human stem cell-derived astrocytes (NCT03482050), while patients affected by retinitis pigmentosa were subject to a dose of glial cells committed to the astrocyte lineage (NCT04284293). An additional trial was scheduled to begin in spring 2021 to investigate the safety of transplanting glial cell precursors in patients with transverse myelitis, a neuroinflammatory disease that can be caused by autoantibodies against the astrocytic protein AQP4 (NCT03887273). No updates have yet been provided on this study. Esposito et al. also explored the possibility of transplanting astrocyte-like enteric glial cells in a rat model of Alzheimer’s disease, thereby showing their capacity to rescue behavioral impairments [96].

In parallel, strategies were pursued to strengthen the supportive properties of the astrocytes. For instance, astrocytes modified to secrete high amounts of L-3,4-dihydroxyphenylalanine (DOPA) were shown to ameliorate the phenotype of a rat model of PD [97]. Furthermore, several papers described the in vivo transplantation of precursors engineered to overexpress the glial cell line-derived neurotrophic factor (GDNF) and their efficacy in ameliorating the pathological phenotype of rodent models of ALS [98,99,100] and PD [101,102]. Based on this, a phase I/IIa clinical trial assessing the safety of GDNF-overexpressing human stem cells committed to astrocyte transplantation in the lumbar spinal cord of ALS patients was later activated (NCT02943850). However, the results have not been released yet. The transplantation of astrocytes genetically modified to overexpress trophic and transcription factors along with neural progenitor cells (NPCs) was tested in a rat model of Parkinson’s disease, where it ameliorated the phenotype by promoting differentiation and survival of NPCs into dopaminergic neurons [88,103]. Related to these approaches, it should be mentioned that they may profit soon from the technological advancements provided by research in biomaterials aiming at boosting the astrocytes neuroprotective properties [104] as well as shielding the implanted cells from the immune system, thereby enhancing their survival [105].

## 5. Modulating Astrocyte Function by Tuning the Incoming Signaling

By virtue of their strategic position within the CNS, astrocytes are particularly prone to integrate signals arising from different neural cell types as well as from the blood vessels. Because microglia-derived cytokines were reported to drive astrocyte conversion into the neurotoxic A1 phenotype, it was initially hypothesized that blocking this process could be a beneficial therapeutic approach to halt neurodegeneration. In keeping with this, the administration of compounds blocking microglia’s ability to elicit A1 astrocytes ameliorated the phenotype, prevented neuron loss, and prolonged survival in mouse models of PD [106] and AD [107]. Furthermore, the concomitant genetic ablation of IL-1α, TNFα, and C1q prevented the emergence of A1 astrocytes and extended survival in a mouse model of ALS [108]. However, in sharp contrast with these data, the same triple knock-out strategy led to the exacerbation of the phenotype in a mouse model of prion diseases [56]. At least two hypotheses were proposed to explain these discrepant results. On the one hand, one might argue that A1 astrocytes are not the one and only subpopulation boosting neurotoxicity in every neurodegenerative condition. On the other hand, it is reasonable to postulate that the constitutive ablation of the three cytokines is an unspecific approach that alters neuroprotective pathways, in addition to blocking the conversion of astrocytes to the A1 status. Globally, this may ultimately lead to a detrimental outcome, while a specific target selection would likely provide more predictable results. In particular, several strategies aiming at stopping pro-inflammatory signaling pathways driving the interactions between microglia and astrocytes improved the phenotypic score in a mouse model of EAE [52]. Although the relevance of these pathways is yet to be confirmed in other neurological conditions, they hold the potential to offer a clean shot to disrupt the building up of a harmful neuroinflammatory milieu.

Astrocytes modulate their functions not only by interacting with microglia-derived factors but also in response to signals arising from endothelial cells, which are major constituents of the BBB. For example, endothelial cells were reported to induce Notch signaling in astrocytes, thereby boosting the expression of the astrocyte-specific glutamate transporter EAAT2 (GLT1 in rodents) [109], a protein involved in the maintenance of extracellular glutamate concentrations below excitotoxic levels and a therapeutic target of outstanding relevance for many neurological conditions. 

During neuropathology, the BBB can become impaired and lead to soluble factors and inflammatory cell infiltration into the perivascular space. Interestingly, in EAE, the cross-talk between endothelial cells and astrocytes was shown to unleash the expression of tight junction molecules in astrocytes, thus restricting the access to the parenchyma of plasmatic proteins and inflammatory cells, as well as limiting the detrimental phenotype in mice [110,111]. Likewise, BBB dysfunction can be also caused by aging, but, in this context, astrocytes respond by hyperactivating the transforming growth factor β (TGFβ) signaling pathway in both rodents and humans. Blocking this signaling cascade in the astrocytes ameliorated cognitive decline and reduced neuronal hyperexcitability in old mice [112].

Ground-breaking studies published in the last few years demonstrated that astrocytes are sensitive to factors of various nature, originating also outside the CNS. In particular, circulating levels of the insulin-like growth factor-1 (IGF-1) were reported to regulate the astrocyte expression of several glutamatergic receptors and the production of eicosanoids, which ensure the optimal neurovascular coupling necessary for intense neuronal activity [113]. Furthermore, the gut microbiota, which can influence many neurological conditions (reviewed in [114]), was shown to release metabolites that tune astrocyte function directly, or through the perturbation of either microglia or meningeal natural killer cells [115,116,117,118,119]. For example, Rothhammer et al. identified an anti-inflammatory effect of tryptophan metabolites, produced by *Lactobacillus Reuteri* and other ampicillin-sensitive gut bacteria [115], even though many aspects of this observation still need to be unraveled. In particular, some of the questions that remain to be addressed are: (i) what is the most favorable probiotic combination to tackle neurodegeneration? (ii) what are the metabolites? (iii) on which cell types do they exert their protective effect? For instance, in a mouse model of ALS, *Akkermansia muciniphila* reduced motor neuron loss and prolonged survival by increasing nicotinamide (vitamin B3) levels, but the mechanisms mediating such positive effects have not yet been resolved at the cellular level [120]. 

Also, calorie restriction was proposed to ameliorate the homeostatic functions of the astrocytes and to improve neuronal plasticity in the mouse hippocampus [121]. By contrast, a high fat, high sugar diet caused astrocytosis and enhanced neurotoxicity in a mouse model of spinal cord injury [119,122]. Finally, physical exercise [123,124,125] and environmental enrichment [123,126,127] importantly contributed to reduce reactive astrocytosis and alleviate the neuroinflammatory response in various animal models of injury and disease. Mechanistically, exercise and dietary restriction are acknowledged means to extend lifespan by triggering several protective pathways in different cells and organs throughout the body. Among others, activation of autophagy emerges as a shared mechanism of action of several life-extending interventions [128], and we can speculate that this occurs also within the CNS. Given the importance of autophagy in neurons [129] as well in astrocytes [130] to protect from neurodegeneration, it would be of outstanding importance to pinpoint the individual contribution of each cell type to refine the most adequate lifestyle intervention. Nevertheless, this evidence supports the view that rehabilitative training could be used to favor morphological remodeling and to improve the functional performance of astrocytes (Figure 1).

Finally, astrocytes have been reported to respond to pesticides and toxins. For example, the herbicide Linuron was shown to target the unfolded protein response in the astrocytes, thereby boosting their neurotoxic role in a mouse model of multiple sclerosis [131]. Furthermore, rats receiving toxin 3-Nitropropionic acid develop A1-type astrocytosis prior to showing neurodegeneration, thereby suggesting that glial cells are the primary targets of this compound [132].

From a therapeutic standpoint, these findings implicate that promoting a healthy lifestyle can positively affect the astrocytes, which, in turn, would perform at their best to ensure optimal CNS function.

## 6. Shared Pathways of Astrocyte Dysfunction in Neurodegenerative Diseases 

Several molecular mechanisms shape the response of the astrocytes to distinct neurodegenerative disorders, such as for example AD [133], ALS [12], HD [134], and PD [135]. Here, we specifically focus on those astrocytic pathways that become dysregulated in multiple conditions. The identification of common pathogenetic signaling cascades is full of implications, as it suggests that therapeutic agents designed to target astrocytes might have a broad spectrum of applications and be an effective add-on therapy. One of the recurring features in multiple pathologies is the loss of the astrocyte-specific glutamate transporter EAAT2, an event that might lead to neurotoxic accumulation of extracellular glutamate (reviewed in [136]). Surprisingly, the antibiotic ceftriaxone was shown to enhance EAAT2 expression [137] and to successfully ameliorate the phenotype of rodent models of different neurodegenerative conditions [138,139,140,141,142,143]. These promising findings led to the prompt activation of clinical trials to evaluate its therapeutic value in ALS (NCT00349622; NCT00718393) and PD dementia (NCT03413384). Regrettably, a Phase III clinical trial failed at demonstrating the efficacy of ceftriaxone in both slowing down disease progression and prolonging survival of ALS patients [144], while the outcome of the PD dementia trial remains to be determined because patients are still being recruited. Another small molecule apt at enhancing EAAT2 expression is LDN/OSU0212320 [145]. Interestingly, the administration of this compound successfully ameliorated the phenotype of rodent models of ALS [145], AD [146], pain [147], migraine [148], and, very recently, stroke, where the beneficial effect was observed in male, but not in female animals [149]. This last observation remarks that not only the development of neurological conditions, but also the response to treatment can be gender-dependent and calls for the design of preclinical and clinical studies taking this option into consideration.

Deregulation of the activity of two transcription factors, namely the nuclear factor erythroid 2-related factor 2 (Nrf2) and the nuclear factor kappa-light-chain-enhancer of activated B cells (NF-kB), is another finding recurring in many neurodegenerative conditions. Although the first controls the expression of antioxidant genes and the second is considered a master regulator of the immune response, their signaling pathways display significant interplays (reviewed in [150]). Intriguingly, Nrf2 is expressed in a wide array of peripheral tissues as well as in the CNS, where its levels are particularly high in the astrocytes. From a functional standpoint, activation of Nrf2 in the astrocytes was reported to powerfully boost their neuroprotective abilities in co-culture systems [151,152]. This evidence prompted a wide array of cellular studies aiming at evaluating the capacity of different chemicals to induce Nrf2 activity in the context of different neurodegenerative disorders, including ALS [152,153,154,155,156], AD [157,158], and PD [159,160]. 

Notably, selective Nrf2 activation in the astrocytes ameliorated the pathological phenotype in animal models of both ALS [161] and PD [162,163], while its genetic ablation worsened the development of EAE in mice [164]. Furthermore, the neuroprotective effect of transplanted astrocytes in a mouse model of PD was reported to rely on their ability to mount an Nrf2-mediated anti-oxidative and pro-survival environment [90]. At variance with this, ALS mice crossed with animals overexpressing Nrf2 in neurons [165] or subjected to neuronal-targeted viral-mediated gene therapy [166] did not exhibit an extended lifespan. Finally, systemic administration of many promising compounds, according to the in vitro studies, had moderate beneficial effects on animal models of ALS [167,168,169], PD [160,170], and AD [158]. Taken together, these discoveries support the view that Nrf2-activating compounds are indeed promising candidates to control several neurodegenerative conditions as long as the therapy is precisely targeted to the astrocytes. Furthermore, a recent study aiming at exploring astrocyte diversity in EAE unveiled that (i) only a subpopulation of astrocytes experiences loss of Nrf2 function with subsequent hyperactivation of the repressor Musculoapoeneuorotic Factor G (MAFG); (ii) this subpopulation is driving the severity of the phenotype; and (iii) genetic ablation of MAFG by viral-mediated gene therapy limits neuroinflammation [164]. In spite of the requirement to specifically target astrocytes, clinical trials have been registered to explore the impact of Nrf2-activating nutritional compounds on the progression of various diseases. In particular, dietary supplementation with sulforaphane, an isothiocyanate found in plants of the *Brassicaceae* family (i.e., cabbage), is under investigation in AD (NCT04213391) and frontal brain damage (NCT04252261) patients. Furthermore, various formulations of curcumin (one of the active compounds of turmeric) are being tested in ALS (NCT04499963; NCT04654689), MS (NCT03150966), cognitive impairment (NCT01383161; NCT01811381) and AD (NCT01001637; NCT00164749; NCT00099710; NCT01811381; NCT01716637; NCT04606420) (Figure 1).

NF-kB is a ubiquitously expressed transcription factor complex that plays a crucial role in the expression of various inflammatory genes. It is widely acknowledged that NF-kB is activated in the astrocytes in the context of several neurodegenerative disorders. However, a point that remains little understood is whether its activation in the astrocytes triggers a neuroprotective or a neurotoxic response. In particular, astrocyte-specific ablation of NF-kB activity was shown to be beneficial in a mouse model of PD [171], as well as in Drosophila models of Spinocerebellar ataxia 3 and AD [172]. Conversely, this approach led to conflicting results in the context of ALS. In murine models of ALS, it was initially noted that preventing NF-kB activation in astrocytes did not ameliorate the pathological phenotype [173,174] or it was even detrimental [175]. In line with these observations, we demonstrated that activating NF-kB in astrocytes can lead to the production of growth factors that are known to support neuronal survival, namely brain-derived neurotrophic factor (BDNF) and GDNF [176]. However, pharmacological treatment with the NF-kB inhibitor Withaferin A ameliorated the phenotype of several models of ALS [177,178,179]. Notably, in astrocyte cultures, Withaferin A prevents the NF-kB-dependent induction of TNFα, a cytokine that strongly activates the transcription factor itself, thereby breaking a self-amplifying vicious cycle [180]. A likely explanation for these apparently discrepant results came from the analysis of the phenotype of ALS mice genetically modified to achieve conditional NF-kB activation in astrocytes upon doxycycline withdrawal. In this model, prolonged NF-kB activation delayed the onset of symptoms, but drove a much faster disease progression and did not impact on survival. On the contrary, a temporary hyperactivation of NF-kB in young mice delayed symptom onset and led to extended lifespan [181]. Taken together, these results suggest that NF-kB activity can produce different outcomes depending on both the cell type and disease stage in which it is modulated. Intriguingly, Kim et al. drew the same conclusions by observing that inhibition of NF-kB signaling in astrocytes prior to the onset of symptoms is detrimental, while at later stages it is beneficial in a mouse model of spinocerebellar ataxia 1 [182].

Finally, it was very recently demonstrated that drugs exhibiting a dual-action, i.e., Nrf2 activation and NF-kB inhibition, display neuroprotective properties in models of AD [158], PD [183,184], and MS [185], thus strengthening the idea that targeting multiple pathways in the astrocytes could correct the aberrant activities of these cells and, consequently, halt neurodegeneration.

## 7. Strategies to Target Astrocytes

The evidence discussed in the previous sections convincingly argues that targeting astrocytes might efficiently counteract the pathogenesis of various neurodegenerative diseases. It also strongly calls for the development of novel delivery tools to convey therapeutic agents of different nature, including nucleic acids, peptides, or small molecules, to the astrocytes. The optimal formulation of these drugs should allow for non-invasive routes of administration and should not show any toxicity. Besides, the therapeutic agent should be able to cross the BBB in order to access the CNS parenchyma to reach an extracellular target or to be selectively internalized by the astrocytes for an intracellular pathway. To achieve these goals, several innovative strategies are currently under development (Figure 1).

### 7.1. Nanoparticles

A particularly flexible formulation to achieve delivery to the CNS is the synthesis and the assembly of nanoparticles (NPs) apt at delivering nucleic acids, proteins, and small molecules [186]. NP is an umbrella term that generically defines artificially assembled structures with a size in the range of 2–500 nm and composed of different types of chemicals. In the context of CNS disorders, the most advanced application of NP technology is in the treatment of brain tumors. For instance, the successful regression of a brain tumor in xenografted mice administered with gold NPs loaded with siRNAs [187] prompted the activation of a clinical trial (NCT03020017) aiming at assessing the safety of this formulation in patients affected by glioblastoma or glioma. Notably, gold accumulation was detected in tumor resected from all treated patients in the absence of overt toxicity, although 25% of patients displayed severe side effects possibly related to the administration of the medicament [188]. Further preclinical development of NPs is ongoing and aims at increasing the available options to deliver cytotoxic chemicals and nucleic acid to tumors, thereby sparing the surrounding homeostatic astrocytes [21,189]. 

However, innovative strategies are necessary to obtain delivery of chemicals specifically to astrocytes in the context of neuroinflammatory and neurodegenerative disorders, ideally upon systemic administration. To achieve these goals, research is expanding in parallel in two directions. On the one hand, NPs can be decorated with different types of peptidic moieties to achieve both the transport across the BBB and the uptake by the astrocytes. For instance, chitosan NPs were functionalized with transferrin and bradykinin B2 receptors to exploit the transcytosis pathway across the BBB and to transduce astrocytes, where they released their siRNA content with the aim of blocking HIV replication [190]. Furthermore, Tanaka et al. developed lipid NPs decorated with ApoE to deliver mRNA and to obtain protein overexpression in both astrocytes and neurons, upon intracerebroventricular administration [191]. Finally, gh6225, a peptide derived from the Herpes Simplex Virus 1, is also valuable as a targeting system across the BBB, alone and upon conjugation with different types of NPs [192,193,194,195]. Notably, the peptide provides good transduction efficiency to both neurons and astrocytes in vitro, while neuronal uptake is predominant in vivo [193]. On the other hand, different types of chemistry allowed obtaining NPs apt at selectively targeting astrocytes and delivering nucleic acids [196,197,198] or small molecules [199]. Surnar et al. developed an even more sophisticated, but promising, strategy by generating NPs with a double targeting system to allow both crossing the BBB upon intravenous administration and delivering their payload to mitochondria. Remarkably, they tested their delivery system in a rat model of ALS, thus demonstrating that NPs targeted to astrocytes reduced the level of reactive oxygen species (ROS) and boosted ATP production in both astrocytes and the neighboring neurons [200]. Finally, dendrimers conjugated with a fluorescent dye displayed increased, but variable, uptake by activated astrocytes in a rat model of cerebral ischemia, thus suggesting that astrocyte diversity can lead to a different endocytosis activity [201]. This finding also implies that, with an accurate choice of different types of chemistry, it will be soon possible to design NPs to selectively target distinct astrocyte subpopulations. 

### 7.2. Peptide-Based Delivery

Cell-permeable peptides are short amino acid sequences, which can cross biological membranes even when coupled with a wide array of therapeutic agents, thereby allowing their intracellular delivery [202]. Interestingly, we showed that a peptide, composed of the BH4 domain of the anti-apoptotic Bcl-xL protein fused to the protein transduction domain of the HIV-1 TAT protein, was able to cross the BBB and protect astrocytes in vivo in a mouse model of ALS, after systemic administration [85]. Although lacking specificity towards astrocytes, this approach resulted promising and highlighted features of the delivery system that are shared by other peptides derived from different viral glycoproteins [193,203,204]. More recently, a homing peptide, named AS1 and showing high affinity and specificity for the astrocytes, was discovered during a phage display study [205]. Furthermore, a cell-penetrating peptide assembled from the phospholipid binding domain of the myristoylated alanine-rich C-kinase substrate and the HIV-1-derived TAT sequence demonstrated intrinsic therapeutic value specifically targeting malignant cells in a mouse model of a brain tumor [206]. 

### 7.3. Viral Delivery

Viruses have co-evolved to selectively invade host cells and take control of their machinery and metabolism to replicate, thus implying that they are natural gene delivery vectors. Consequently, many classes of viruses have been taken into consideration to develop gene therapy paradigms to treat human diseases (http://www.abedia.com/wiley/, accessed on 5 August 2021). However, gene therapy for CNS disorders has long suffered from the lack of a suitable vector apt at crossing the BBB upon systemic administration. This major pitfall was overcome in 2009 when Foust et al. discovered that the adeno-associated virus serotype 9 (AAV9) mainly transduces neurons when administered to neonate mice, while it infects also astrocytes in adult animals [207] and in non-human primates [208]. This promising result prompted additional investigations aiming at increasing the specificity of gene transfer to the astrocytes by either modifying the genome vector and/or by developing engineered capsids to increase the tropism towards astrocytes. In particular, cell-specific gene expression was achieved using astrocyte-specific promoters [209,210,211,212,213,214,215,216,217,218,219]. In parallel, screening strategies led to the identification of cell-type-specific capsids able to enhance the transduction efficiency of the astrocytes by recombinant vectors, namely AAV Anc80L65 [220] and AAV-PHP.B [221,222,223], when compared to AAV9. From a therapeutic standpoint, it is actually debated whether AAV-PHP.B displays analogous tropism in non-human primates [224,225,226]. A complementary approach to modulate AAV tropism to astrocytes consists in producing chimeric AAV capsids that display synthetic peptides on the surface of wild-type capsids. These are, for example, the cases of AAV9P1 [227] and MNM017, which, at least in vitro, efficiently infect human astrocytes [228].

In spite of their excellent safety profile, the reduced cloning allowance (~4.7 kb for conventional viruses and only ~2.3 kb for self-complementary viruses) greatly limits the application of AAV to gene therapy. To overcome this issue, the portfolio of viral vectors includes also lentiviruses, which are specialized retroviruses with a ~10 kb cloning capacity. Lentiviruses can be assembled using different envelope glycoproteins, a technique known as “pseudotyping”, which represents a very effective way to modulate their tropism. Notably, lentiviruses pseudotyped with glycoproteins from the lymphocytic choriomeningitis virus, the Moloney murine leukemia virus [229], the Mokola virus [230,231], or the chikungunya virus [232] were reported to acquire tropism towards astrocytes. As discussed in the context of AAV development, the use of an astrocyte-specific promoter is also valuable to achieve cell-specific gene transfer, mainly with the addition of a miRNA de-targeting strategy to abolish residual transgene expression in neuronal cells [230,233,234].

## 8. Astrocyte Role in Adult Neurogenesis and as Neuronal Precursors

Adult neurogenesis is a process occurring in specialized compartments of the subventricular zone and the subgranular zone of the hippocampal dentate gyrus, known as neurogenic niches, and contributes to important physiological CNS functions, such as learning and memory [235]. Notably, astrocytes as well as other glial cell types contribute to the maintenance of a permissive microenvironment for neurogenesis within the niche [236]. During aging and neurodegenerative conditions, such as PD [237] and AD [238], adult neurogenesis is impaired and contributes to the pathogenesis of the disease. A possible explanation for this dysfunction is that the astrocytes lose their ability to sustain neurogenesis. In keeping with this, in a mouse model of AD, aberrant accumulation of a tau isoform in dentate gyrus astrocytes was reported to hamper adult neurogenesis and spatial memory [239], thus suggesting that selectively targeting astrocytes within the niche may restore neurogenesis and, possibly, tackle disease progression. 

A complementary intriguing option that is recently coming to the spotlight is to restore lost functions by exploiting the potential of the astrocytes to trans-differentiate into neurons. In particular, it has been shown that forced expression of selected transcription factors can transform astrocytes into glutamatergic [240,241,242,243,244], GABAergic [243,245,246], dopaminergic [247], retinal [248,249], and motor neurons [250] in mice. Intriguingly, astrocyte trans-differentiation into neurons can be achieved also by downregulating the expression of the polypyrimidine tract binding protein 1 gene (*Ptbp1*) by either a short hairpin RNA [251] or by expression of an ortholog of CRISPR-Cas13d (CasRx) and two suitable guide RNAs [252]. Both approaches were reported to reverse the phenotype of a mouse model of PD. These trans-differentiation studies also unveiled another interesting property of the astrocytes, i.e., the capacity of replacing neurons they were interacting with. More specifically, in a mouse model of cortical stab wound injury, the overexpression of two transcription factors, namely nuclear receptor-related 1 protein (Nurr1) and Neurogenin 2 (Ngn2), in astrocytes from different cortical layers prompted their conversion into neurons that expressed markers that were consistent with their laminar localization [241]. In keeping with this, viral delivery of *CasRx* to ablate the expression of Ptbp1 in the retina converted Müller glial cells into retinal neurons, while in the striatum it led to the generation of dopaminergic neurons [252]. It is important to mention that, although these results have not yet been replicated in non-human primates, they provide promising perspectives in terms of reverting brain and spinal cord functions that are lost in neurodegenerative disorders.

## 9. Conclusions

In the past years, our knowledge of the biology of astrocytes as well as of their contribution to the pathogenesis of many neurological disorders has increased exponentially. Furthermore, the blossoming of sequencing techniques has provided molecular evidence of the extreme heterogeneity of the astrocytes, an aspect that was previously acknowledged only through early morphological descriptions. The increased comprehension of the features and properties of these cells offers unprecedented therapeutic opportunities: it is becoming more and more evident that to achieve control over neurodegeneration, it is essential to modulate the activities of distinct astrocyte subpopulations at different stages of the disease. Additional in-depth investigations are certainly necessary in order to achieve this goal. The rapidly growing development of bioinformatic skills, including artificial intelligence, will likely enable a truly unbiased interpretation of the large datasets generated by sequencing experiments, thereby uncovering the most relevant astrocytic de-regulated pathways (reviewed in [32]). Moreover, because we are still unable to specifically deliver drugs to distinct astrocyte subpopulations, major research investments are necessary to enable the development of innovative tools to improve the discharge of chemicals and nucleic acids precisely to astrocytes over neurons or other glial cell types. Additionally, the actual protocols aiming at replacing astrocytes cannot control which specific subpopulation is re-installed and should be, therefore, refined to allow the generation of specific astrocytic phenotypes. Finally, it is highly likely that pharmacological, genetic, or cell therapies should be complemented by lifestyle interventions, including the intake of diets rich in BBB-permeable antioxidant nutrients, the control of the gut flora, and the execution of physical exercise, to improve the astrocyte performance and, consequently, to slow down the neurodegenerative process.

## Data Availability

Not applicable.

## References

[1] Hou Y., Dan X., Babbar M., Wei Y., Hasselbalch S.G., Croteau D.L., Bohr V.A. (2019). Ageing as a risk factor for neurodegenerative disease. Nat. Rev. Neurol..

[2] Rossi D. (2015). Astrocyte physiopathology: At the crossroads of intercellular networking, inflammation and cell death. Prog. Neurobiol..

[3] Verkhratsky A., Parpura V., Vardjan N., Zorec R. (2019). Physiology of Astroglia. Adv. Exp. Med. Biol..

[4] Langen U.H., Ayloo S., Gu C. (2019). Development and Cell Biology of the Blood-Brain Barrier. Annu. Rev. Cell. Dev. Biol..

[5] Mestre H., Mori Y., Nedergaard M. (2020). The Brain’s Glymphatic System: Current Controversies. Trends Neurosci..

[6] Namekawa M., Takiyama Y., Aoki Y., Takayashiki N., Sakoe K., Shimazaki H., Taguchi T., Tanaka Y., Nishizawa M., Saito K. (2002). Identification of GFAP gene mutation in hereditary adult-onset Alexander’s disease. Ann. Neurol..

[7] Jarius S., Paul F., Weinshenker B.G., Levy M., Kim H.J., Wildemann B. (2020). Neuromyelitis optica. Nat. Rev. Dis. Prim..

[8] Tavcar P., Potokar M., Kolenc M., Korva M., Avsic-Zupanc T., Zorec R., Jorgacevski J. (2021). Neurotropic Viruses, Astrocytes, and COVID-19. Front. Cell. Neurosci..

[9] Han X., Zhang T., Liu H., Mi Y., Gou X. (2020). Astrocyte Senescence and Alzheimer’s Disease: A Review. Front. Aging Neurosci..

[10] Miyazaki I., Asanuma M. (2020). Neuron-Astrocyte Interactions in Parkinson’s Disease. Cells.

[11] Wilton D.K., Stevens B. (2020). The contribution of glial cells to Huntington’s disease pathogenesis. Neurobiol. Dis..

[12] Valori C.F., Guidotti G., Brambilla L., Rossi D. (2019). Astrocytes in Motor Neuron Diseases. Adv. Exp. Med. Biol..

[13] Sofroniew M.V. (2020). Astrocyte Reactivity: Subtypes, States, and Functions in CNS Innate Immunity. Trends Immunol..

[14] Valori C.F., Guidotti G., Brambilla L., Rossi D. (2019). Astrocytes: Emerging Therapeutic Targets in Neurological Disorders. Trends Mol. Med..

[15] Tsai H.H., Li H., Fuentealba L.C., Molofsky A.V., Taveira-Marques R., Zhuang H., Tenney A., Murnen A.T., Fancy S.P., Merkle F. (2012). Regional astrocyte allocation regulates CNS synaptogenesis and repair. Science.

[16] Molofsky A.V., Kelley K.W., Tsai H.H., Redmond S.A., Chang S.M., Madireddy L., Chan J.R., Baranzini S.E., Ullian E.M., Rowitch D.H. (2014). Astrocyte-encoded positional cues maintain sensorimotor circuit integrity. Nature.

[17] Morel L., Chiang M.S.R., Higashimori H., Shoneye T., Iyer L.K., Yelick J., Tai A., Yang Y. (2017). Molecular and Functional Properties of Regional Astrocytes in the Adult Brain. J. Neurosci..

[18] Farmer W.T., Abrahamsson T., Chierzi S., Lui C., Zaelzer C., Jones E.V., Bally B.P., Chen G.G., Theroux J.F., Peng J. (2016). Neurons diversify astrocytes in the adult brain through sonic hedgehog signaling. Science.

[19] Lanjakornsiripan D., Pior B.J., Kawaguchi D., Furutachi S., Tahara T., Katsuyama Y., Suzuki Y., Fukazawa Y., Gotoh Y. (2018). Layer-specific morphological and molecular differences in neocortical astrocytes and their dependence on neuronal layers. Nat. Commun..

[20] Bayraktar O.A., Bartels T., Holmqvist S., Kleshchevnikov V., Martirosyan A., Polioudakis D., Ben Haim L., Young A.M.H., Batiuk M.Y., Prakash K. (2020). Astrocyte layers in the mammalian cerebral cortex revealed by a single-cell in situ transcriptomic map. Nat. Neurosci..

[21] Azambuja J.H., Schuh R.S., Michels L.R., Gelsleichter N.E., Beckenkamp L.R., Iser I.C., Lenz G.S., de Oliveira F.H., Venturin G., Greggio S. (2020). Nasal Administration of Cationic Nanoemulsions as CD73-siRNA Delivery System for Glioblastoma Treatment: A New Therapeutical Approach. Mol. Neurobiol..

[22] Stahlberg A., Andersson D., Aurelius J., Faiz M., Pekna M., Kubista M., Pekny M. (2011). Defining cell populations with single-cell gene expression profiling: Correlations and identification of astrocyte subpopulations. Nucleic Acids Res..

[23] Zeisel A., Munoz-Manchado A.B., Codeluppi S., Lonnerberg P., La Manno G., Jureus A., Marques S., Munguba H., He L., Betsholtz C. (2015). Brain structure. Cell types in the mouse cortex and hippocampus revealed by single-cell RNA-seq. Science.

[24] Gokce O., Stanley G.M., Treutlein B., Neff N.F., Camp J.G., Malenka R.C., Rothwell P.E., Fuccillo M.V., Sudhof T.C., Quake S.R. (2016). Cellular Taxonomy of the Mouse Striatum as Revealed by Single-Cell RNA-Seq. Cell Rep..

[25] Zeisel A., Hochgerner H., Lonnerberg P., Johnsson A., Memic F., van der Zwan J., Haring M., Braun E., Borm L.E., La Manno G. (2018). Molecular Architecture of the Mouse Nervous System. Cell.

[26] Zhu Q., Shah S., Dries R., Cai L., Yuan G.C. (2018). Identification of spatially associated subpopulations by combining scRNAseq and sequential fluorescence in situ hybridization data. Nat. Biotechnol..

[27] Batiuk M.Y., Martirosyan A., Wahis J., de Vin F., Marneffe C., Kusserow C., Koeppen J., Viana J.F., Oliveira J.F., Voet T. (2020). Identification of region-specific astrocyte subtypes at single cell resolution. Nat. Commun..

[28] Habib N., McCabe C., Medina S., Varshavsky M., Kitsberg D., Dvir-Szternfeld R., Green G., Dionne D., Nguyen L., Marshall J.L. (2020). Disease-associated astrocytes in Alzheimer’s disease and aging. Nat. Neurosci..

[29] Lu T., Mar J.C. (2020). Investigating transcriptome-wide sex dimorphism by multi-level analysis of single-cell RNA sequencing data in ten mouse cell types. Biol. Sex Differ..

[30] Rurak G.M., Simard S., Charih F., Van Geel A., Stead J., Woodside B., Green J.R., Coppola G., Salmaso N. (2020). Translatomic database of cortical astroglia across male and female mouse development reveals two distinct developmental phenotypes. bioRxiv.

[31] Chai H., Diaz-Castro B., Shigetomi E., Monte E., Octeau J.C., Yu X., Cohn W., Rajendran P.S., Vondriska T.M., Whitelegge J.P. (2017). Neural Circuit-Specialized Astrocytes: Transcriptomic, Proteomic, Morphological, and Functional Evidence. Neuron.

[32] Myszczynska M.A., Ojamies P.N., Lacoste A.M.B., Neil D., Saffari A., Mead R., Hautbergue G.M., Holbrook J.D., Ferraiuolo L. (2020). Applications of machine learning to diagnosis and treatment of neurodegenerative diseases. Nat. Rev. Neurol..

[33] Patterson K.C., Kahanovitch U., Goncalves C.M., Hablitz J.J., Staruschenko A., Mulkey D.K., Olsen M.L. (2021). Kir 5.1-dependent CO2/H(+) -sensitive currents contribute to astrocyte heterogeneity across brain regions. Glia.

[34] Crespo-Castrillo A., Garcia-Segura L.M., Arevalo M.A. (2020). The synthetic steroid tibolone exerts sex-specific regulation of astrocyte phagocytosis under basal conditions and after an inflammatory challenge. J. Neuroinflamm..

[35] Ibrahim M.M.H., Bheemanapally K., Sylvester P.W., Briski K.P. (2020). Sex-specific estrogen regulation of hypothalamic astrocyte estrogen receptor expression and glycogen metabolism in rats. Mol. Cell. Endocrinol..

[36] Oberheim N.A., Takano T., Han X., He W., Lin J.H., Wang F., Xu Q., Wyatt J.D., Pilcher W., Ojemann J.G. (2009). Uniquely hominid features of adult human astrocytes. J. Neurosci..

[37] Zhang Y., Sloan S.A., Clarke L.E., Caneda C., Plaza C.A., Blumenthal P.D., Vogel H., Steinberg G.K., Edwards M.S., Li G. (2016). Purification and Characterization of Progenitor and Mature Human Astrocytes Reveals Transcriptional and Functional Differences with Mouse. Neuron.

[38] Kelley K.W., Nakao-Inoue H., Molofsky A.V., Oldham M.C. (2018). Variation among intact tissue samples reveals the core transcriptional features of human CNS cell classes. Nat. Neurosci..

[39] Hodge R.D., Bakken T.E., Miller J.A., Smith K.A., Barkan E.R., Graybuck L.T., Close J.L., Long B., Johansen N., Penn O. (2019). Conserved cell types with divergent features in human versus mouse cortex. Nature.

[40] Li L., Shi Y. (2020). When glia meet induced pluripotent stem cells (iPSCs). Mol. Cell. Neurosci..

[41] Bradley R.A., Shireman J., McFalls C., Choi J., Canfield S.G., Dong Y., Liu K., Lisota B., Jones J.R., Petersen A. (2019). Regionally specified human pluripotent stem cell-derived astrocytes exhibit different molecular signatures and functional properties. Development.

[42] Gatto N., Dos Santos Souza C., Shaw A.C., Bell S.M., Myszczynska M.A., Powers S., Meyer K., Castelli L.M., Karyka E., Mortiboys H. (2021). Directly converted astrocytes retain the ageing features of the donor fibroblasts and elucidate the astrocytic contribution to human CNS health and disease. Aging Cell.

[43] Marton R.M., Pasca S.P. (2020). Organoid and Assembloid Technologies for Investigating Cellular Crosstalk in Human Brain Development and Disease. Trends Cell Biol..

[44] Dang J., Tiwari S.K., Agrawal K., Hui H., Qin Y., Rana T.M. (2021). Glial cell diversity and methamphetamine-induced neuroinflammation in human cerebral organoids. Mol. Psychiatry.

[45] Liddelow S.A., Marsh S.E., Stevens B. (2020). Microglia and Astrocytes in Disease: Dynamic Duo or Partners in Crime?. Trends Immunol..

[46] Giovannoni F., Quintana F.J. (2020). The Role of Astrocytes in CNS Inflammation. Trends Immunol..

[47] Linnerbauer M., Rothhammer V. (2020). Protective Functions of Reactive Astrocytes Following Central Nervous System Insult. Front. Immunol..

[48] Linnerbauer M., Wheeler M.A., Quintana F.J. (2020). Astrocyte Crosstalk in CNS Inflammation. Neuron.

[49] Zamanian J.L., Xu L., Foo L.C., Nouri N., Zhou L., Giffard R.G., Barres B.A. (2012). Genomic analysis of reactive astrogliosis. J. Neurosci..

[50] Liddelow S.A., Guttenplan K.A., Clarke L.E., Bennett F.C., Bohlen C.J., Schirmer L., Bennett M.L., Munch A.E., Chung W.S., Peterson T.C. (2017). Neurotoxic reactive astrocytes are induced by activated microglia. Nature.

[51] Joshi A.U., Minhas P.S., Liddelow S.A., Haileselassie B., Andreasson K.I., Dorn G.W., Mochly-Rosen D. (2019). Fragmented mitochondria released from microglia trigger A1 astrocytic response and propagate inflammatory neurodegeneration. Nat. Neurosci..

[52] Clark I.C., Gutierrez-Vazquez C., Wheeler M.A., Li Z., Rothhammer V., Linnerbauer M., Sanmarco L.M., Guo L., Blain M., Zandee S.E.J. (2021). Barcoded viral tracing of single-cell interactions in central nervous system inflammation. Science.

[53] Damisah E.C., Hill R.A., Rai A., Chen F., Rothlin C.V., Ghosh S., Grutzendler J. (2020). Astrocytes and microglia play orchestrated roles and respect phagocytic territories during neuronal corpse removal in vivo. Sci. Adv..

[54] Boisvert M.M., Erikson G.A., Shokhirev M.N., Allen N.J. (2018). The Aging Astrocyte Transcriptome from Multiple Regions of the Mouse Brain. Cell Rep..

[55] Clarke L.E., Liddelow S.A., Chakraborty C., Munch A.E., Heiman M., Barres B.A. (2018). Normal aging induces A1-like astrocyte reactivity. Proc. Natl. Acad. Sci. USA.

[56] Hartmann K., Sepulveda-Falla D., Rose I.V.L., Madore C., Muth C., Matschke J., Butovsky O., Liddelow S., Glatzel M., Krasemann S. (2019). Complement 3(+)-astrocytes are highly abundant in prion diseases, but their abolishment led to an accelerated disease course and early dysregulation of microglia. Acta Neuropathol. Commun..

[57] Ugalde C.L., Lewis V., Stehmann C., McLean C.A., Lawson V.A., Collins S.J., Hill A.F. (2020). Markers of A1 astrocytes stratify to molecular sub-types in sporadic Creutzfeldt-Jakob disease brain. Brain Commun..

[58] Diaz-Castro B., Gangwani M.R., Yu X., Coppola G., Khakh B.S. (2019). Astrocyte molecular signatures in Huntington’s disease. Sci. Transl. Med..

[59] Al-Dalahmah O., Sosunov A.A., Shaik A., Ofori K., Liu Y., Vonsattel J.P., Adorjan I., Menon V., Goldman J.E. (2020). Single-nucleus RNA-seq identifies Huntington disease astrocyte states. Acta Neuropathol. Commun..

[60] Sun S., Sun Y., Ling S.C., Ferraiuolo L., McAlonis-Downes M., Zou Y., Drenner K., Wang Y., Ditsworth D., Tokunaga S. (2015). Translational profiling identifies a cascade of damage initiated in motor neurons and spreading to glia in mutant SOD1-mediated ALS. Proc. Natl. Acad. Sci. USA.

[61] Sekar S., McDonald J., Cuyugan L., Aldrich J., Kurdoglu A., Adkins J., Serrano G., Beach T.G., Craig D.W., Valla J. (2015). Alzheimer’s disease is associated with altered expression of genes involved in immune response and mitochondrial processes in astrocytes. Neurobiol. Aging.

[62] Wu T., Dejanovic B., Gandham V.D., Gogineni A., Edmonds R., Schauer S., Srinivasan K., Huntley M.A., Wang Y., Wang T.M. (2019). Complement C3 Is Activated in Human AD Brain and Is Required for Neurodegeneration in Mouse Models of Amyloidosis and Tauopathy. Cell Rep..

[63] Smith H.L., Freeman O.J., Butcher A.J., Holmqvist S., Humoud I., Schatzl T., Hughes D.T., Verity N.C., Swinden D.P., Hayes J. (2020). Astrocyte Unfolded Protein Response Induces a Specific Reactivity State that Causes Non-Cell-Autonomous Neuronal Degeneration. Neuron.

[64] Yu X., Nagai J., Marti-Solano M., Soto J.S., Coppola G., Babu M.M., Khakh B.S. (2020). Context-Specific Striatal Astrocyte Molecular Responses Are Phenotypically Exploitable. Neuron.

[65] Verkhratsky A., Augusto-Oliveira M., Pivoriunas A., Popov A., Brazhe A., Semyanov A. (2021). Astroglial asthenia and loss of function, rather than reactivity, contribute to the ageing of the brain. Pflügers Arch. Eur. J. Physiol..

[66] Liddelow S.A., Barres B.A. (2017). Reactive Astrocytes: Production, Function, and Therapeutic Potential. Immunity.

[67] Escartin C., Galea E., Lakatos A., O’Callaghan J.P., Petzold G.C., Serrano-Pozo A., Steinhauser C., Volterra A., Carmignoto G., Agarwal A. (2021). Reactive astrocyte nomenclature, definitions, and future directions. Nat. Neurosci..

[68] Lennol M.P., Canelles S., Guerra-Cantera S., Argente J., Garcia-Segura L.M., de Ceballos M.L., Chowen J.A., Frago L.M. (2021). Amyloid-beta1-40 differentially stimulates proliferation, activation of oxidative stress and inflammatory responses in male and female hippocampal astrocyte cultures. Mech. Ageing Dev..

[69] Tassoni A., Farkhondeh V., Itoh Y., Itoh N., Sofroniew M.V., Voskuhl R.R. (2019). The astrocyte transcriptome in EAE optic neuritis shows complement activation and reveals a sex difference in astrocytic C3 expression. Sci. Rep..

[70] Chowen J.A., Garcia-Segura L.M. (2021). Role of glial cells in the generation of sex differences in neurodegenerative diseases and brain aging. Mech. Ageing Dev..

[71] Lananna B.V., Nadarajah C.J., Izumo M., Cedeno M.R., Xiong D.D., Dimitry J., Tso C.F., McKee C.A., Griffin P., Sheehan P.W. (2018). Cell-Autonomous Regulation of Astrocyte Activation by the Circadian Clock Protein BMAL1. Cell Rep..

[72] Yilmaz C., Karali K., Fodelianaki G., Gravanis A., Chavakis T., Charalampopoulos I., Alexaki V.I. (2019). Neurosteroids as regulators of neuroinflammation. Front. Neuroendocrinol..

[73] Tamai T.K., Nakane Y., Ota W., Kobayashi A., Ishiguro M., Kadofusa N., Ikegami K., Yagita K., Shigeyoshi Y., Sudo M. (2018). Identification of circadian clock modulators from existing drugs. EMBO Mol. Med..

[74] Barca-Mayo O., Boender A.J., Armirotti A., De Pietri Tonelli D. (2020). Deletion of astrocytic BMAL1 results in metabolic imbalance and shorter lifespan in mice. Glia.

[75] Barca-Mayo O., Pons-Espinal M., Follert P., Armirotti A., Berdondini L., De Pietri Tonelli D. (2017). Astrocyte deletion of Bmal1 alters daily locomotor activity and cognitive functions via GABA signalling. Nat. Commun..

[76] Carter B., Justin H.S., Gulick D., Gamsby J.J. (2021). The Molecular Clock and Neurodegenerative Disease: A Stressful Time. Front. Mol. Biosci..

[77] Sase S., Takanohashi A., Vanderver A., Almad A. (2018). Astrocytes, an active player in Aicardi-Goutieres syndrome. Brain Pathol..

[78] Rodriguez J.J., Olabarria M., Chvatal A., Verkhratsky A. (2009). Astroglia in dementia and Alzheimer’s disease. Cell Death Differ..

[79] Saha P., Biswas S.C. (2015). Amyloid-beta induced astrocytosis and astrocyte death: Implication of FoxO3a-Bim-caspase3 death signaling. Mol. Cell. Neurosci..

[80] Ramos-Gonzalez P., Mato S., Chara J.C., Verkhratsky A., Matute C., Cavaliere F. (2021). Astrocytic atrophy as a pathological feature of Parkinson’s disease with LRRK2 mutation. NPJ Parkinsons Dis..

[81] Bedner P., Dupper A., Huttmann K., Muller J., Herde M.K., Dublin P., Deshpande T., Schramm J., Haussler U., Haas C.A. (2015). Astrocyte uncoupling as a cause of human temporal lobe epilepsy. Brain.

[82] Fan H., Zhang K., Shan L., Kuang F., Chen K., Zhu K., Ma H., Ju G., Wang Y.Z. (2016). Reactive astrocytes undergo M1 microglia/macrohpages-induced necroptosis in spinal cord injury. Mol. Neurodegener..

[83] Ni Y., Gu W.W., Liu Z.H., Zhu Y.M., Rong J.G., Kent T.A., Li M., Qiao S.G., An J.Z., Zhang H.L. (2018). RIP1K Contributes to Neuronal and Astrocytic Cell Death in Ischemic Stroke via Activating Autophagic-lysosomal Pathway. Neuroscience.

[84] Rossi D., Brambilla L., Valori C.F., Roncoroni C., Crugnola A., Yokota T., Bredesen D.E., Volterra A. (2008). Focal degeneration of astrocytes in amyotrophic lateral sclerosis. Cell Death Differ..

[85] Martorana F., Brambilla L., Valori C.F., Bergamaschi C., Roncoroni C., Aronica E., Volterra A., Bezzi P., Rossi D. (2012). The BH4 domain of Bcl-X(L) rescues astrocyte degeneration in amyotrophic lateral sclerosis by modulating intracellular calcium signals. Hum. Mol. Genet..

[86] Komine O., Yamashita H., Fujimori-Tonou N., Koike M., Jin S., Moriwaki Y., Endo F., Watanabe S., Uematsu S., Akira S. (2018). Innate immune adaptor TRIF deficiency accelerates disease progression of ALS mice with accumulation of aberrantly activated astrocytes. Cell Death Differ..

[87] Pihlaja R., Koistinaho J., Malm T., Sikkila H., Vainio S., Koistinaho M. (2008). Transplanted astrocytes internalize deposited beta-amyloid peptides in a transgenic mouse model of Alzheimer’s disease. Glia.

[88] Proschel C., Stripay J.L., Shih C.H., Munger J.C., Noble M.D. (2014). Delayed transplantation of precursor cell-derived astrocytes provides multiple benefits in a rat model of Parkinsons. EMBO Mol. Med..

[89] Sun Y., Lu X.J., Fu X., Zhang Y., Zhan Y., Liu J., Zhao L., Xia C.L. (2021). Engrafted primary type-2 astrocytes improve the recovery of the nigrostriatal pathway in a rat model of Parkinson’s disease. Mol. Cell. Biochem..

[90] Serapide M.F., L’Episcopo F., Tirolo C., Testa N., Caniglia S., Giachino C., Marchetti B. (2020). Boosting Antioxidant Self-defenses by Grafting Astrocytes Rejuvenates the Aged Microenvironment and Mitigates Nigrostriatal Toxicity in Parkinsonian Brain via an *Nrf2*-Driven Wnt/beta-Catenin Prosurvival Axis. Front. Aging Neurosci..

[91] Lepore A.C., Rauck B., Dejea C., Pardo A.C., Rao M.S., Rothstein J.D., Maragakis N.J. (2008). Focal transplantation-based astrocyte replacement is neuroprotective in a model of motor neuron disease. Nat. Neurosci..

[92] Kondo T., Funayama M., Tsukita K., Hotta A., Yasuda A., Nori S., Kaneko S., Nakamura M., Takahashi R., Okano H. (2014). Focal transplantation of human iPSC-derived glial-rich neural progenitors improves lifespan of ALS mice. Stem Cell Rep..

[93] Izrael M., Slutsky S.G., Admoni T., Cohen L., Granit A., Hasson A., Itskovitz-Eldor J., Krush Paker L., Kuperstein G., Lavon N. (2018). Safety and efficacy of human embryonic stem cell-derived astrocytes following intrathecal transplantation in SOD1(G93A) and NSG animal models. Stem Cell Res. Ther..

[94] Goldberg N.R.S., Marsh S.E., Ochaba J., Shelley B.C., Davtyan H., Thompson L.M., Steffan J.S., Svendsen C.N., Blurton-Jones M. (2017). Human Neural Progenitor Transplantation Rescues Behavior and Reduces alpha-Synuclein in a Transgenic Model of Dementia with Lewy Bodies. Stem Cells Transl. Med..

[95] Llorente I.L., Xie Y., Mazzitelli J.A., Hatanaka E.A., Cinkornpumin J., Miller D.R., Lin Y., Lowry W.E., Carmichael S.T. (2021). Patient-derived glial enriched progenitors repair functional deficits due to white matter stroke and vascular dementia in rodents. Sci. Transl. Med..

[96] Esposito G., Sarnelli G., Capoccia E., Cirillo C., Pesce M., Lu J., Cali G., Cuomo R., Steardo L. (2016). Autologous transplantation of intestine-isolated glia cells improves neuropathology and restores cognitive deficits in beta amyloid-induced neurodegeneration. Sci. Rep..

[97] Lundberg C., Horellou P., Mallet J., Bjorklund A. (1996). Generation of DOPA-producing astrocytes by retroviral transduction of the human tyrosine hydroxylase gene: In vitro characterization and in vivo effects in the rat Parkinson model. Exp. Neurol..

[98] Klein S.M., Behrstock S., McHugh J., Hoffmann K., Wallace K., Suzuki M., Aebischer P., Svendsen C.N. (2005). GDNF delivery using human neural progenitor cells in a rat model of ALS. Hum. Gene Ther..

[99] Thomsen G.M., Avalos P., Ma A.A., Alkaslasi M., Cho N., Wyss L., Vit J.P., Godoy M., Suezaki P., Shelest O. (2018). Transplantation of Neural Progenitor Cells Expressing Glial Cell Line-Derived Neurotrophic Factor into the Motor Cortex as a Strategy to Treat Amyotrophic Lateral Sclerosis. Stem Cells.

[100] Akhtar A.A., Gowing G., Kobritz N., Savinoff S.E., Garcia L., Saxon D., Cho N., Kim G., Tom C.M., Park H. (2018). Inducible Expression of GDNF in Transplanted iPSC-Derived Neural Progenitor Cells. Stem Cell Rep..

[101] Cunningham L.A., Su C. (2002). Astrocyte delivery of glial cell line-derived neurotrophic factor in a mouse model of Parkinson’s disease. Exp. Neurol..

[102] Ericson C., Georgievska B., Lundberg C. (2005). Ex vivo gene delivery of GDNF using primary astrocytes transduced with a lentiviral vector provides neuroprotection in a rat model of Parkinson’s disease. Eur. J. Neurosci..

[103] Song J.J., Oh S.M., Kwon O.C., Wulansari N., Lee H.S., Chang M.Y., Lee E., Sun W., Lee S.E., Chang S. (2018). Cografting astrocytes improves cell therapeutic outcomes in a Parkinson’s disease model. J. Clin. Investig..

[104] Kim B.J., Choi J.Y., Choi H., Han S., Seo J., Kim J., Joo S., Kim H.M., Oh C., Hong S. (2020). Astrocyte-Encapsulated Hydrogel Microfibers Enhance Neuronal Circuit Generation. Adv. Healthc. Mater..

[105] Kuddannaya S., Zhu W., Chu C., Singh A., Walczak P., Bulte J.W.M. (2021). In Vivo Imaging of Allografted Glial-Restricted Progenitor Cell Survival and Hydrogel Scaffold Biodegradation. ACS Appl. Mater. Interfaces.

[106] Yun S.P., Kam T.I., Panicker N., Kim S., Oh Y., Park J.S., Kwon S.H., Park Y.J., Karuppagounder S.S., Park H. (2018). Block of A1 astrocyte conversion by microglia is neuroprotective in models of Parkinson’s disease. Nat. Med..

[107] Guo M.F., Zhang H.Y., Li Y.H., Gu Q.F., Wei W.Y., Wang Y.Y., Zhang X.J., Liu X.Q., Song L.J., Chai Z. (2020). Fasudil inhibits the activation of microglia and astrocytes of transgenic Alzheimer’s disease mice via the downregulation of TLR4/Myd88/NF-kappaB pathway. J. Neuroimmunol..

[108] Guttenplan K.A., Weigel M.K., Adler D.I., Couthouis J., Liddelow S.A., Gitler A.D., Barres B.A. (2020). Knockout of reactive astrocyte activating factors slows disease progression in an ALS mouse model. Nat. Commun..

[109] Lee M.L., Martinez-Lozada Z., Krizman E.N., Robinson M.B. (2017). Brain endothelial cells induce astrocytic expression of the glutamate transporter GLT-1 by a Notch-dependent mechanism. J. Neurochem..

[110] Horng S., Therattil A., Moyon S., Gordon A., Kim K., Argaw A.T., Hara Y., Mariani J.N., Sawai S., Flodby P. (2017). Astrocytic tight junctions control inflammatory CNS lesion pathogenesis. J. Clin. Investig..

[111] Mora P., Hollier P.L., Guimbal S., Abelanet A., Diop A., Cornuault L., Couffinhal T., Horng S., Gadeau A.P., Renault M.A. (2020). Blood-brain barrier genetic disruption leads to protective barrier formation at the Glia Limitans. PLoS Biol..

[112] Senatorov V.V., Friedman A.R., Milikovsky D.Z., Ofer J., Saar-Ashkenazy R., Charbash A., Jahan N., Chin G., Mihaly E., Lin J.M. (2019). Blood-brain barrier dysfunction in aging induces hyperactivation of TGFbeta signaling and chronic yet reversible neural dysfunction. Sci. Transl. Med..

[113] Toth P., Tarantini S., Ashpole N.M., Tucsek Z., Milne G.L., Valcarcel-Ares N.M., Menyhart A., Farkas E., Sonntag W.E., Csiszar A. (2015). IGF-1 deficiency impairs neurovascular coupling in mice: Implications for cerebromicrovascular aging. Aging Cell.

[114] Needham B.D., Kaddurah-Daouk R., Mazmanian S.K. (2020). Gut microbial molecules in behavioural and neurodegenerative conditions. Nat. Rev. Neurosci..

[115] Rothhammer V., Mascanfroni I.D., Bunse L., Takenaka M.C., Kenison J.E., Mayo L., Chao C.C., Patel B., Yan R., Blain M. (2016). Type I interferons and microbial metabolites of tryptophan modulate astrocyte activity and central nervous system inflammation via the aryl hydrocarbon receptor. Nat. Med..

[116] Rothhammer V., Borucki D.M., Tjon E.C., Takenaka M.C., Chao C.C., Ardura-Fabregat A., de Lima K.A., Gutierrez-Vazquez C., Hewson P., Staszewski O. (2018). Microglial control of astrocytes in response to microbial metabolites. Nature.

[117] Margineanu M.B., Sherwin E., Golubeva A., Peterson V., Hoban A., Fiumelli H., Rea K., Cryan J.F., Magistretti P.J. (2020). Gut microbiota modulates expression of genes involved in the astrocyte-neuron lactate shuttle in the hippocampus. Eur. Neuropsychopharmacol..

[118] Sanmarco L.M., Wheeler M.A., Gutierrez-Vazquez C., Polonio C.M., Linnerbauer M., Pinho-Ribeiro F.A., Li Z., Giovannoni F., Batterman K.V., Scalisi G. (2021). Gut-licensed IFNgamma(+) NK cells drive LAMP1(+)TRAIL(+) anti-inflammatory astrocytes. Nature.

[119] Heiss C.N., Manneras-Holm L., Lee Y.S., Serrano-Lobo J., Hakansson Gladh A., Seeley R.J., Drucker D.J., Backhed F., Olofsson L.E. (2021). The gut microbiota regulates hypothalamic inflammation and leptin sensitivity in Western diet-fed mice via a GLP-1R-dependent mechanism. Cell Rep..

[120] Blacher E., Bashiardes S., Shapiro H., Rothschild D., Mor U., Dori-Bachash M., Kleimeyer C., Moresi C., Harnik Y., Zur M. (2019). Potential roles of gut microbiome and metabolites in modulating ALS in mice. Nature.

[121] Popov A., Denisov P., Bychkov M., Brazhe A., Lyukmanova E., Shenkarev Z., Lazareva N., Verkhratsky A., Semyanov A. (2020). Caloric restriction triggers morphofunctional remodeling of astrocytes and enhances synaptic plasticity in the mouse hippocampus. Cell Death Dis..

[122] Kim H.N., Langley M.R., Simon W.L., Yoon H., Kleppe L., Lanza I.R., LeBrasseur N.K., Matveyenko A., Scarisbrick I.A. (2020). A Western diet impairs CNS energy homeostasis and recovery after spinal cord injury: Link to astrocyte metabolism. Neurobiol. Dis..

[123] Rodriguez J.J., Terzieva S., Olabarria M., Lanza R.G., Verkhratsky A. (2013). Enriched environment and physical activity reverse astrogliodegeneration in the hippocampus of AD transgenic mice. Cell Death Dis..

[124] Lundquist A.J., Parizher J., Petzinger G.M., Jakowec M.W. (2019). Exercise induces region-specific remodeling of astrocyte morphology and reactive astrocyte gene expression patterns in male mice. J. Neurosci. Res..

[125] Yin M., Pu T., Wang L., Marshall C., Wu T., Xiao M. (2018). Astroglial water channel aquaporin 4-mediated glymphatic clearance function: A determined factor for time-sensitive treatment of aerobic exercise in patients with Alzheimer’s disease. Med. Hypotheses.

[126] Sampedro-Piquero P., De Bartolo P., Petrosini L., Zancada-Menendez C., Arias J.L., Begega A. (2014). Astrocytic plasticity as a possible mediator of the cognitive improvements after environmental enrichment in aged rats. Neurobiol. Learn Mem..

[127] Diniz D.G., de Oliveira M.A., de Lima C.M., Foro C.A., Sosthenes M.C., Bento-Torres J., da Costa Vasconcelos P.F., Anthony D.C., Diniz C.W. (2016). Age, environment, object recognition and morphological diversity of GFAP-immunolabeled astrocytes. Behav. Brain Funct..

[128] Bareja A., Lee D.E., White J.P. (2019). Maximizing Longevity and Healthspan: Multiple Approaches All Converging on Autophagy. Front. Cell. Dev. Biol..

[129] Menzies F.M., Fleming A., Caricasole A., Bento C.F., Andrews S.P., Ashkenazi A., Fullgrabe J., Jackson A., Jimenez Sanchez M., Karabiyik C. (2017). Autophagy and Neurodegeneration: Pathogenic Mechanisms and Therapeutic Opportunities. Neuron.

[130] Sung K., Jimenez-Sanchez M. (2020). Autophagy in Astrocytes and its Implications in Neurodegeneration. J. Mol. Biol..

[131] Wheeler M.A., Jaronen M., Covacu R., Zandee S.E.J., Scalisi G., Rothhammer V., Tjon E.C., Chao C.C., Kenison J.E., Blain M. (2019). Environmental Control of Astrocyte Pathogenic Activities in CNS Inflammation. Cell.

[132] Lopez-Sanchez C., Garcia-Martinez V., Poejo J., Garcia-Lopez V., Salazar J., Gutierrez-Merino C. (2020). Early Reactive A1 Astrocytes Induction by the Neurotoxin 3-Nitropropionic Acid in Rat Brain. Int. J. Mol. Sci..

[133] Preman P., Alfonso-Triguero M., Alberdi E., Verkhratsky A., Arranz A.M. (2021). Astrocytes in Alzheimer’s Disease: Pathological Significance and Molecular Pathways. Cells.

[134] Gray M. (2019). Astrocytes in Huntington’s Disease. Adv. Exp. Med. Biol..

[135] Udovin L., Quarracino C., Herrera M.I., Capani F., Otero-Losada M., Perez-Lloret S. (2020). Role of Astrocytic Dysfunction in the Pathogenesis of Parkinson’s Disease Animal Models from a Molecular Signaling Perspective. Neural Plast..

[136] Todd A.C., Hardingham G.E. (2020). The Regulation of Astrocytic Glutamate Transporters in Health and Neurodegenerative Diseases. Int. J. Mol. Sci..

[137] Rothstein J.D., Patel S., Regan M.R., Haenggeli C., Huang Y.H., Bergles D.E., Jin L., Dykes Hoberg M., Vidensky S., Chung D.S. (2005). Beta-lactam antibiotics offer neuroprotection by increasing glutamate transporter expression. Nature.

[138] Zumkehr J., Rodriguez-Ortiz C.J., Cheng D., Kieu Z., Wai T., Hawkins C., Kilian J., Lim S.L., Medeiros R., Kitazawa M. (2015). Ceftriaxone ameliorates tau pathology and cognitive decline via restoration of glial glutamate transporter in a mouse model of Alzheimer’s disease. Neurobiol. Aging.

[139] Miller B.R., Dorner J.L., Shou M., Sari Y., Barton S.J., Sengelaub D.R., Kennedy R.T., Rebec G.V. (2008). Up-regulation of GLT1 expression increases glutamate uptake and attenuates the Huntington’s disease phenotype in the R6/2 mouse. Neuroscience.

[140] Chotibut T., Davis R.W., Arnold J.C., Frenchek Z., Gurwara S., Bondada V., Geddes J.W., Salvatore M.F. (2014). Ceftriaxone increases glutamate uptake and reduces striatal tyrosine hydroxylase loss in 6-OHDA Parkinson’s model. Mol. Neurobiol..

[141] Ho S.C., Hsu C.C., Pawlak C.R., Tikhonova M.A., Lai T.J., Amstislavskaya T.G., Ho Y.J. (2014). Effects of ceftriaxone on the behavioral and neuronal changes in an MPTP-induced Parkinson’s disease rat model. Behav. Brain. Res..

[142] Bisht R., Kaur B., Gupta H., Prakash A. (2014). Ceftriaxone mediated rescue of nigral oxidative damage and motor deficits in MPTP model of Parkinson’s disease in rats. Neurotoxicology.

[143] Weng J.C., Tikhonova M.A., Chen J.H., Shen M.S., Meng W.Y., Chang Y.T., Chen K.H., Liang K.C., Hung C.S., Amstislavskaya T.G. (2016). Ceftriaxone prevents the neurodegeneration and decreased neurogenesis seen in a Parkinson’s disease rat model: An immunohistochemical and MRI study. Behav. Brain Res..

[144] Cudkowicz M.E., Titus S., Kearney M., Yu H., Sherman A., Schoenfeld D., Hayden D., Shui A., Brooks B., Conwit R. (2014). Safety and efficacy of ceftriaxone for amyotrophic lateral sclerosis: A multi-stage, randomised, double-blind, placebo-controlled trial. Lancet Neurol..

[145] Kong Q., Chang L.C., Takahashi K., Liu Q., Schulte D.A., Lai L., Ibabao B., Lin Y., Stouffer N., Das Mukhopadhyay C. (2014). Small-molecule activator of glutamate transporter EAAT2 translation provides neuroprotection. J. Clin. Investig..

[146] Takahashi K., Kong Q., Lin Y., Stouffer N., Schulte D.A., Lai L., Liu Q., Chang L.C., Dominguez S., Xing X. (2015). Restored glial glutamate transporter EAAT2 function as a potential therapeutic approach for Alzheimer’s disease. J. Exp. Med..

[147] Alotaibi G., Rahman S. (2019). Effects of glial glutamate transporter activator in formalin-induced pain behaviour in mice. Eur. J. Pain.

[148] Zhou X., Liang J., Wang J., Fei Z., Qin G., Zhang D., Zhou J., Chen L. (2020). Up-regulation of astrocyte excitatory amino acid transporter 2 alleviates central sensitization in a rat model of chronic migraine. J. Neurochem..

[149] Tejeda-Bayron F.A., Rivera-Aponte D.E., Malpica-Nieves C.J., Maldonado-Martinez G., Maldonado H.M., Skatchkov S.N., Eaton M.J. (2021). Activation of Glutamate Transporter-1 (GLT-1) Confers Sex-Dependent Neuroprotection in Brain Ischemia. Brain Sci..

[150] Sivandzade F., Prasad S., Bhalerao A., Cucullo L. (2019). NRF2 and NF-B interplay in cerebrovascular and neurodegenerative disorders: Molecular mechanisms and possible therapeutic approaches. Redox Biol..

[151] Shih A.Y., Johnson D.A., Wong G., Kraft A.D., Jiang L., Erb H., Johnson J.A., Murphy T.H. (2003). Coordinate regulation of glutathione biosynthesis and release by *Nrf2*-expressing glia potently protects neurons from oxidative stress. J. Neurosci..

[152] Vargas M.R., Pehar M., Cassina P., Martinez-Palma L., Thompson J.A., Beckman J.S., Barbeito L. (2005). Fibroblast growth factor-1 induces heme oxygenase-1 via nuclear factor erythroid 2-related factor 2 (*Nrf2*) in spinal cord astrocytes: Consequences for motor neuron survival. J. Biol. Chem..

[153] Diaz-Amarilla P., Miquel E., Trostchansky A., Trias E., Ferreira A.M., Freeman B.A., Cassina P., Barbeito L., Vargas M.R., Rubbo H. (2016). Electrophilic nitro-fatty acids prevent astrocyte-mediated toxicity to motor neurons in a cell model of familial amyotrophic lateral sclerosis via nuclear factor erythroid 2-related factor activation. Free Radic. Biol. Med..

[154] Vargas M.R., Pehar M., Cassina P., Beckman J.S., Barbeito L. (2006). Increased glutathione biosynthesis by *Nrf2* activation in astrocytes prevents p75NTR-dependent motor neuron apoptosis. J. Neurochem..

[155] Hoang T.T., Johnson D.A., Raines R.T., Johnson J.A. (2019). Angiogenin activates the astrocytic *Nrf2*/antioxidant-response element pathway and thereby protects murine neurons from oxidative stress. J. Biol. Chem..

[156] Harlan B.A., Pehar M., Killoy K.M., Vargas M.R. (2019). Enhanced SIRT6 activity abrogates the neurotoxic phenotype of astrocytes expressing ALS-linked mutant SOD1. FASEB J..

[157] Oksanen M., Hyotylainen I., Trontti K., Rolova T., Wojciechowski S., Koskuvi M., Viitanen M., Levonen A.L., Hovatta I., Roybon L. (2020). NF-E2-related factor 2 activation boosts antioxidant defenses and ameliorates inflammatory and amyloid properties in human Presenilin-1 mutated Alzheimer’s disease astrocytes. Glia.

[158] Ikram M., Muhammad T., Rehman S.U., Khan A., Jo M.G., Ali T., Kim M.O. (2019). Hesperetin Confers Neuroprotection by Regulating *Nrf2*/TLR4/NF-kappaB Signaling in an Abeta Mouse Model. Mol. Neurobiol..

[159] Izumi Y., Kataoka H., Inose Y., Akaike A., Koyama Y., Kume T. (2018). Neuroprotective effect of an *Nrf2*-ARE activator identified from a chemical library on dopaminergic neurons. Eur. J. Pharmacol..

[160] Park J.S., Leem Y.H., Park J.E., Kim D.Y., Kim H.S. (2019). Neuroprotective Effect of beta-Lapachone in MPTP-Induced Parkinson’s Disease Mouse Model: Involvement of Astroglial p-AMPK/*Nrf2*/HO-1 Signaling Pathways. Biomol. Ther. (Seoul).

[161] Vargas M.R., Johnson D.A., Sirkis D.W., Messing A., Johnson J.A. (2008). *Nrf2* activation in astrocytes protects against neurodegeneration in mouse models of familial amyotrophic lateral sclerosis. J. Neurosci..

[162] Chen P.C., Vargas M.R., Pani A.K., Smeyne R.J., Johnson D.A., Kan Y.W., Johnson J.A. (2009). *Nrf2*-mediated neuroprotection in the MPTP mouse model of Parkinson’s disease: Critical role for the astrocyte. Proc. Natl. Acad. Sci. USA.

[163] Wei Y., Lu M., Mei M., Wang H., Han Z., Chen M., Yao H., Song N., Ding X., Ding J. (2020). Pyridoxine induces glutathione synthesis via PKM2-mediated *Nrf2* transactivation and confers neuroprotection. Nat. Commun..

[164] Wheeler M.A., Clark I.C., Tjon E.C., Li Z., Zandee S.E.J., Couturier C.P., Watson B.R., Scalisi G., Alkwai S., Rothhammer V. (2020). MAFG-driven astrocytes promote CNS inflammation. Nature.

[165] Vargas M.R., Burton N.C., Kutzke J., Gan L., Johnson D.A., Schafer M., Werner S., Johnson J.A. (2013). Absence of *Nrf2* or its selective overexpression in neurons and muscle does not affect survival in ALS-linked mutant hSOD1 mouse models. PLoS ONE.

[166] Nanou A., Higginbottom A., Valori C.F., Wyles M., Ning K., Shaw P., Azzouz M. (2013). Viral delivery of antioxidant genes as a therapeutic strategy in experimental models of amyotrophic lateral sclerosis. Mol. Ther..

[167] Feng X., Peng Y., Liu M., Cui L. (2012). DL-3-n-butylphthalide extends survival by attenuating glial activation in a mouse model of amyotrophic lateral sclerosis. Neuropharmacology.

[168] Mead R.J., Higginbottom A., Allen S.P., Kirby J., Bennett E., Barber S.C., Heath P.R., Coluccia A., Patel N., Gardner I. (2013). S[+] Apomorphine is a CNS penetrating activator of the *Nrf2*-ARE pathway with activity in mouse and patient fibroblast models of amyotrophic lateral sclerosis. Free Radic. Biol. Med..

[169] Neymotin A., Calingasan N.Y., Wille E., Naseri N., Petri S., Damiano M., Liby K.T., Risingsong R., Sporn M., Beal M.F. (2011). Neuroprotective effect of *Nrf2*/ARE activators, CDDO ethylamide and CDDO trifluoroethylamide, in a mouse model of amyotrophic lateral sclerosis. Free Radic. Biol. Med..

[170] Inose Y., Izumi Y., Takada-Takatori Y., Akaike A., Koyama Y., Kaneko S., Kume T. (2020). Protective effects of *Nrf2*-ARE activator on dopaminergic neuronal loss in Parkinson disease model mice: Possible involvement of heme oxygenase-1. Neurosci. Lett..

[171] Hammond S.L., Bantle C.M., Popichak K.A., Wright K.A., Thompson D., Forero C., Kirkley K.S., Damale P.U., Chong E.K.P., Tjalkens R.B. (2020). NF-kappaB Signaling in Astrocytes Modulates Brain Inflammation and Neuronal Injury Following Sequential Exposure to Manganese and MPTP During Development and Aging. Toxicol. Sci..

[172] Li Y.X., Sibon O.C.M., Dijkers P.F. (2018). Inhibition of NF-kappaB in astrocytes is sufficient to delay neurodegeneration induced by proteotoxicity in neurons. J. Neuroinflamm..

[173] Crosio C., Valle C., Casciati A., Iaccarino C., Carri M.T. (2011). Astroglial inhibition of NF-kappaB does not ameliorate disease onset and progression in a mouse model for amyotrophic lateral sclerosis (ALS). PLoS ONE.

[174] Frakes A.E., Ferraiuolo L., Haidet-Phillips A.M., Schmelzer L., Braun L., Miranda C.J., Ladner K.J., Bevan A.K., Foust K.D., Godbout J.P. (2014). Microglia induce motor neuron death via the classical NF-kappaB pathway in amyotrophic lateral sclerosis. Neuron.

[175] Lee J.Y., Lee J.D., Phipps S., Noakes P.G., Woodruff T.M. (2015). Absence of toll-like receptor 4 (TLR4) extends survival in the hSOD1 G93A mouse model of amyotrophic lateral sclerosis. J. Neuroinflamm..

[176] Brambilla L., Martorana F., Guidotti G., Rossi D. (2018). Dysregulation of Astrocytic HMGB1 Signaling in Amyotrophic Lateral Sclerosis. Front. Neurosci..

[177] Patel P., Julien J.P., Kriz J. (2015). Early-stage treatment with Withaferin A reduces levels of misfolded superoxide dismutase 1 and extends lifespan in a mouse model of amyotrophic lateral sclerosis. Neurotherapeutics.

[178] Swarup V., Phaneuf D., Dupre N., Petri S., Strong M., Kriz J., Julien J.P. (2011). Deregulation of TDP-43 in amyotrophic lateral sclerosis triggers nuclear factor kappaB-mediated pathogenic pathways. J. Exp. Med..

[179] Kumar S., Phaneuf D., Julien J.P. (2021). Withaferin-A Treatment Alleviates TAR DNA-Binding Protein-43 Pathology and Improves Cognitive Function in a Mouse Model of FTLD. Neurotherapeutics.

[180] Martorana F., Guidotti G., Brambilla L., Rossi D. (2015). Withaferin A Inhibits Nuclear Factor-kappaB-Dependent Pro-Inflammatory and Stress Response Pathways in the Astrocytes. Neural Plast..

[181] Ouali Alami N., Schurr C., Olde Heuvel F., Tang L., Li Q., Tasdogan A., Kimbara A., Nettekoven M., Ottaviani G., Raposo C. (2018). NF-kappaB activation in astrocytes drives a stage-specific beneficial neuroimmunological response in ALS. EMBO J..

[182] Kim J.H., Lukowicz A., Qu W., Johnson A., Cvetanovic M. (2018). Astroglia contribute to the pathogenesis of spinocerebellar ataxia Type 1 (SCA1) in a biphasic, stage-of-disease specific manner. Glia.

[183] Yang Y., Kong F., Ding Q., Cai Y., Hao Y., Tang B. (2020). Bruceine D elevates *Nrf2* activation to restrain Parkinson’s disease in mice through suppressing oxidative stress and inflammatory response. Biochem. Biophys. Res. Commun..

[184] Jo M.G., Ikram M., Jo M.H., Yoo L., Chung K.C., Nah S.Y., Hwang H., Rhim H., Kim M.O. (2019). Gintonin Mitigates MPTP-Induced Loss of Nigrostriatal Dopaminergic Neurons and Accumulation of alpha-Synuclein via the *Nrf2*/HO-1 Pathway. Mol. Neurobiol..

[185] Colombo E., Bassani C., De Angelis A., Ruffini F., Ottoboni L., Comi G., Martino G., Farina C. (2020). Siponimod (BAF312) Activates *Nrf2* While Hampering NFkappaB in Human Astrocytes, and Protects From Astrocyte-Induced Neurodegeneration. Front. Immunol..

[186] Thangudu S., Cheng F.Y., Su C.H. (2020). Advancements in the Blood-Brain Barrier Penetrating Nanoplatforms for Brain Related Disease Diagnostics and Therapeutic Applications. Polymers.

[187] Jensen S.A., Day E.S., Ko C.H., Hurley L.A., Luciano J.P., Kouri F.M., Merkel T.J., Luthi A.J., Patel P.C., Cutler J.I. (2013). Spherical nucleic acid nanoparticle conjugates as an RNAi-based therapy for glioblastoma. Sci. Transl. Med..

[188] Kumthekar P., Rademaker A., Ko C., Dixit K., Schwartz M.A., Sonabend A.M., Sharp L., Lukas R.V., Stupp R., Horbinski C. (2019). A phase 0 first-in-human study using NU-0129: A gold base spherical nucleic acid (SNA) nanoconjugate targeting BCL2L12 in recurrent glioblastoma patients. J. Clin. Oncol..

[189] Lee J.A., Ayat N., Sun Z., Tofilon P.J., Lu Z.R., Camphausen K. (2020). Improving Radiation Response in Glioblastoma Using ECO/siRNA Nanoparticles Targeting DNA Damage Repair. Cancers.

[190] Gu J., Al-Bayati K., Ho E.A. (2017). Development of antibody-modified chitosan nanoparticles for the targeted delivery of siRNA across the blood-brain barrier as a strategy for inhibiting HIV replication in astrocytes. Drug Deliv. Transl. Res..

[191] Tanaka H., Nakatani T., Furihata T., Tange K., Nakai Y., Yoshioka H., Harashima H., Akita H. (2018). In Vivo Introduction of mRNA Encapsulated in Lipid Nanoparticles to Brain Neuronal Cells and Astrocytes via Intracerebroventricular Administration. Mol. Pharm..

[192] Guarnieri D., Falanga A., Muscetti O., Tarallo R., Fusco S., Galdiero M., Galdiero S., Netti P.A. (2013). Shuttle-mediated nanoparticle delivery to the blood-brain barrier. Small.

[193] Valiante S., Falanga A., Cigliano L., Iachetta G., Busiello R.A., La Marca V., Galdiero M., Lombardi A., Galdiero S. (2015). Peptide gH625 enters into neuron and astrocyte cell lines and crosses the blood-brain barrier in rats. Int. J. Nanomed..

[194] Falanga A., Iachetta G., Lombardi L., Perillo E., Lombardi A., Morelli G., Valiante S., Galdiero S. (2018). Enhanced uptake of gH625 by blood brain barrier compared to liver in vivo: Characterization of the mechanism by an in vitro model and implications for delivery. Sci. Rep..

[195] Iachetta G., Falanga A., Molino Y., Masse M., Jabes F., Mechioukhi Y., Laforgia V., Khrestchatisky M., Galdiero S., Valiante S. (2019). gH625-liposomes as tool for pituitary adenylate cyclase-activating polypeptide brain delivery. Sci. Rep..

[196] Joshi C.R., Raghavan V., Vijayaraghavalu S., Gao Y., Saraswathy M., Labhasetwar V., Ghorpade A. (2018). Reaching for the Stars in the Brain: Polymer-Mediated Gene Delivery to Human Astrocytes. Mol. Ther. Nucleic. Acids.

[197] Proulx J., Joshi C., Vijayaraghavalu S., Saraswathy M., Labhasetwar V., Ghorpade A., Borgmann K. (2020). Arginine-Modified Polymers Facilitate Poly (Lactide-Co-Glycolide)-Based Nanoparticle Gene Delivery to Primary Human Astrocytes. Int. J. Nanomed..

[198] Fatima N., Gromnicova R., Loughlin J., Sharrack B., Male D. (2020). Gold nanocarriers for transport of oligonucleotides across brain endothelial cells. PLoS ONE.

[199] Papa S., Veneruso V., Mauri E., Cremonesi G., Mingaj X., Mariani A., De Paola M., Rossetti A., Sacchetti A., Rossi F. (2020). Functionalized nanogel for treating activated astrocytes in spinal cord injury. J. Control. Release.

[200] Surnar B., Basu U., Banik B., Ahmad A., Marples B., Kolishetti N., Dhar S. (2018). Nanotechnology-mediated crossing of two impermeable membranes to modulate the stars of the neurovascular unit for neuroprotection. Proc. Natl. Acad. Sci. USA.

[201] Murta V., Schilrreff P., Rosciszewski G., Morilla M.J., Ramos A.J. (2018). G5G2.5 core-shell tecto-dendrimer specifically targets reactive glia in brain ischemia. J. Neurochem..

[202] Guidotti G., Brambilla L., Rossi D. (2020). Exploring Novel Molecular Targets for the Treatment of High-Grade Astrocytomas Using Peptide Therapeutics: An Overview. Cells.

[203] Villa-Cedillo S.A., Rodriguez-Rocha H., Zavala-Flores L.M., Montes-de-Oca-Luna R., Garcia-Garcia A., Loera-Arias M.J., Saucedo-Cardenas O. (2017). Asn194Lys mutation in RVG29 peptide increases GFP transgene delivery by endocytosis to neuroblastoma and astrocyte cells. J. Pharm. Pharmacol..

[204] Villa-Cedillo S.A., Soto-Dominguez A., Rodriguez-Rocha H., Garcia-Garcia A., de Jesus Loera-Arias M., Rivera-Chavez L.F., Acosta-Espinoza E.J., Valdes J., Zavala-Flores L.M., Montes-de-Oca-Luna R. (2019). The mRVG-9R peptide as a potential therapeutic vector to the central nervous system cells. Cell Biol. Int..

[205] Terashima T., Ogawa N., Nakae Y., Sato T., Katagi M., Okano J., Maegawa H., Kojima H. (2018). Gene Therapy for Neuropathic Pain through siRNA-IRF5 Gene Delivery with Homing Peptides to Microglia. Mol. Ther. Nucleic. Acids.

[206] Eustace N.J., Anderson J.C., Warram J.M., Widden H.N., Pedersen R.T., Alrefai H., Patel Z., Hicks P.H., Placzek W.J., Gillespie G.Y. (2020). A cell-penetrating MARCKS mimetic selectively triggers cytolytic death in glioblastoma. Oncogene.

[207] Foust K.D., Nurre E., Montgomery C.L., Hernandez A., Chan C.M., Kaspar B.K. (2009). Intravascular AAV9 preferentially targets neonatal neurons and adult astrocytes. Nat. Biotechnol..

[208] Samaranch L., Salegio E.A., San Sebastian W., Kells A.P., Foust K.D., Bringas J.R., Lamarre C., Forsayeth J., Kaspar B.K., Bankiewicz K.S. (2012). Adeno-associated virus serotype 9 transduction in the central nervous system of nonhuman primates. Hum. Gene Ther..

[209] Ortinski P.I., Dong J., Mungenast A., Yue C., Takano H., Watson D.J., Haydon P.G., Coulter D.A. (2010). Selective induction of astrocytic gliosis generates deficits in neuronal inhibition. Nat. Neurosci..

[210] von Jonquieres G., Mersmann N., Klugmann C.B., Harasta A.E., Lutz B., Teahan O., Housley G.D., Frohlich D., Kramer-Albers E.M., Klugmann M. (2013). Glial promoter selectivity following AAV-delivery to the immature brain. PLoS ONE.

[211] Meng X., Yang F., Ouyang T., Liu B., Wu C., Jiang W. (2015). Specific gene expression in mouse cortical astrocytes is mediated by a 1740bp-GFAP promoter-driven combined adeno-associated virus 2/5/7/8/9. Neurosci. Lett..

[212] Vagner T., Dvorzhak A., Wojtowicz A.M., Harms C., Grantyn R. (2016). Systemic application of AAV vectors targeting GFAP-expressing astrocytes in Z-Q175-KI Huntington’s disease mice. Mol. Cell. Neurosci..

[213] Taschenberger G., Tereshchenko J., Kugler S. (2017). A MicroRNA124 Target Sequence Restores Astrocyte Specificity of gfaABC1D-Driven Transgene Expression in AAV-Mediated Gene Transfer. Mol. Ther. Nucleic. Acids.

[214] Griffin J.M., Fackelmeier B., Fong D.M., Mouravlev A., Young D., O’Carroll S.J. (2019). Astrocyte-selective AAV gene therapy through the endogenous GFAP promoter results in robust transduction in the rat spinal cord following injury. Gene. Ther..

[215] Pajarillo E., Johnson J., Rizor A., Nyarko-Danquah I., Adinew G., Bornhorst J., Stiboller M., Schwerdtle T., Son D.S., Aschner M. (2020). Astrocyte-specific deletion of the transcription factor Yin Yang 1 in murine substantia nigra mitigates manganese-induced dopaminergic neurotoxicity. J. Biol. Chem..

[216] Koh W., Park Y.M., Lee S.E., Lee C.J. (2017). AAV-Mediated Astrocyte-Specific Gene Expression under Human ALDH1L1 Promoter in Mouse Thalamus. Exp. Neurobiol..

[217] Mudannayake J.M., Mouravlev A., Fong D.M., Young D. (2016). Transcriptional activity of novel ALDH1L1 promoters in the rat brain following AAV vector-mediated gene transfer. Mol. Ther. Methods Clin. Dev..

[218] Birolini G., Verlengia G., Talpo F., Maniezzi C., Zentilin L., Giacca M., Conforti P., Cordiglieri C., Caccia C., Leoni V. (2021). SREBP2 gene therapy targeting striatal astrocytes ameliorates Huntington’s disease phenotypes. Brain.

[219] Guo L., Gao T., Gao C., Jia X., Ni J., Han C., Wang Y. (2021). Stimulation of astrocytic sigma-1 receptor is sufficient to ameliorate inflammation- induced depression. Behav. Brain Res..

[220] Hudry E., Andres-Mateos E., Lerner E.P., Volak A., Cohen O., Hyman B.T., Maguire C.A., Vandenberghe L.H. (2018). Efficient Gene Transfer to the Central Nervous System by Single-Stranded Anc80L65. Mol. Ther. Methods Clin. Dev..

[221] Deverman B.E., Pravdo P.L., Simpson B.P., Kumar S.R., Chan K.Y., Banerjee A., Wu W.L., Yang B., Huber N., Pasca S.P. (2016). Cre-dependent selection yields AAV variants for widespread gene transfer to the adult brain. Nat. Biotechnol..

[222] Chan K.Y., Jang M.J., Yoo B.B., Greenbaum A., Ravi N., Wu W.L., Sanchez-Guardado L., Lois C., Mazmanian S.K., Deverman B.E. (2017). Engineered AAVs for efficient noninvasive gene delivery to the central and peripheral nervous systems. Nat. Neurosci..

[223] Rincon M.Y., de Vin F., Duque S.I., Fripont S., Castaldo S.A., Bouhuijzen-Wenger J., Holt M.G. (2018). Widespread transduction of astrocytes and neurons in the mouse central nervous system after systemic delivery of a self-complementary AAV-PHP.B vector. Gene. Ther..

[224] Matsuzaki Y., Konno A., Mochizuki R., Shinohara Y., Nitta K., Okada Y., Hirai H. (2018). Intravenous administration of the adeno-associated virus-PHP.B capsid fails to upregulate transduction efficiency in the marmoset brain. Neurosci. Lett..

[225] Hordeaux J., Wang Q., Katz N., Buza E.L., Bell P., Wilson J.M. (2018). The Neurotropic Properties of AAV-PHP.B Are Limited to C57BL/6J Mice. Mol. Ther..

[226] Liguore W.A., Domire J.S., Button D., Wang Y., Dufour B.D., Srinivasan S., McBride J.L. (2019). AAV-PHP.B Administration Results in a Differential Pattern of CNS Biodistribution in Non-human Primates Compared with Mice. Mol. Ther..

[227] Kunze C., Borner K., Kienle E., Orschmann T., Rusha E., Schneider M., Radivojkov-Blagojevic M., Drukker M., Desbordes S., Grimm D. (2018). Synthetic AAV/CRISPR vectors for blocking HIV-1 expression in persistently infected astrocytes. Glia.

[228] Davidsson M., Wang G., Aldrin-Kirk P., Cardoso T., Nolbrant S., Hartnor M., Mudannayake J., Parmar M., Bjorklund T. (2019). A systematic capsid evolution approach performed in vivo for the design of AAV vectors with tailored properties and tropism. Proc. Natl. Acad. Sci. USA.

[229] Cannon J.R., Sew T., Montero L., Burton E.A., Greenamyre J.T. (2011). Pseudotype-dependent lentiviral transduction of astrocytes or neurons in the rat substantia nigra. Exp. Neurol..

[230] Colin A., Faideau M., Dufour N., Auregan G., Hassig R., Andrieu T., Brouillet E., Hantraye P., Bonvento G., Deglon N. (2009). Engineered lentiviral vector targeting astrocytes in vivo. Glia.

[231] Pertusa M., Garcia-Matas S., Mammeri H., Adell A., Rodrigo T., Mallet J., Cristofol R., Sarkis C., Sanfeliu C. (2008). Expression of GDNF transgene in astrocytes improves cognitive deficits in aged rats. Neurobiol. Aging.

[232] Eleftheriadou I., Dieringer M., Poh X.Y., Sanchez-Garrido J., Gao Y., Sgourou A., Simmons L.E., Mazarakis N.D. (2017). Selective transduction of astrocytic and neuronal CNS subpopulations by lentiviral vectors pseudotyped with Chikungunya virus envelope. Biomaterials.

[233] Merienne N., Delzor A., Viret A., Dufour N., Rey M., Hantraye P., Deglon N. (2015). Gene transfer engineering for astrocyte-specific silencing in the CNS. Gene. Ther..

[234] Humbel M., Ramosaj M., Zimmer V., Regio S., Aeby L., Moser S., Boizot A., Sipion M., Rey M., Deglon N. (2021). Maximizing lentiviral vector gene transfer in the CNS. Gene. Ther..

[235] Vicidomini C., Guo N., Sahay A. (2020). Communication, Cross Talk, and Signal Integration in the Adult Hippocampal Neurogenic Niche. Neuron.

[236] Araki T., Ikegaya Y., Koyama R. (2020). The effects of microglia-and astrocyte-derived factors on neurogenesis in health and disease. Eur. J. Neurosci..

[237] Marchetti B., Tirolo C., L’Episcopo F., Caniglia S., Testa N., Smith J.A., Pluchino S., Serapide M.F. (2020). Parkinson’s disease, aging and adult neurogenesis: Wnt/beta-catenin signalling as the key to unlock the mystery of endogenous brain repair. Aging Cell.

[238] Casse F., Richetin K., Toni N. (2018). Astrocytes’ Contribution to Adult Neurogenesis in Physiology and Alzheimer’s Disease. Front. Cell. Neurosci..

[239] Richetin K., Steullet P., Pachoud M., Perbet R., Parietti E., Maheswaran M., Eddarkaoui S., Begard S., Pythoud C., Rey M. (2020). Tau accumulation in astrocytes of the dentate gyrus induces neuronal dysfunction and memory deficits in Alzheimer’s disease. Nat. Neurosci..

[240] Berninger B., Costa M.R., Koch U., Schroeder T., Sutor B., Grothe B., Gotz M. (2007). Functional properties of neurons derived from in vitro reprogrammed postnatal astroglia. J. Neurosci..

[241] Mattugini N., Bocchi R., Scheuss V., Russo G.L., Torper O., Lao C.L., Gotz M. (2019). Inducing Different Neuronal Subtypes from Astrocytes in the Injured Mouse Cerebral Cortex. Neuron.

[242] Puls B., Ding Y., Zhang F., Pan M., Lei Z., Pei Z., Jiang M., Bai Y., Forsyth C., Metzger M. (2020). Regeneration of Functional Neurons After Spinal Cord Injury via in situ NeuroD1-Mediated Astrocyte-to-Neuron Conversion. Front. Cell Dev. Biol..

[243] Liu F., Zhang Y., Chen F., Yuan J., Li S., Han S., Lu D., Geng J., Rao Z., Sun L. (2021). Neurog2 directly converts astrocytes into functional neurons in midbrain and spinal cord. Cell Death Dis..

[244] Jiang M.Q., Yu S.P., Wei Z.Z., Zhong W., Cao W., Gu X., Wu A., McCrary M.R., Berglund K., Wei L. (2021). Conversion of Reactive Astrocytes to Induced Neurons Enhances Neuronal Repair and Functional Recovery After Ischemic Stroke. Front. Aging Neurosci..

[245] Heinrich C., Blum R., Gascon S., Masserdotti G., Tripathi P., Sanchez R., Tiedt S., Schroeder T., Gotz M., Berninger B. (2010). Directing astroglia from the cerebral cortex into subtype specific functional neurons. PLoS Biol..

[246] Liu Y., Miao Q., Yuan J., Han S., Zhang P., Li S., Rao Z., Zhao W., Ye Q., Geng J. (2015). Ascl1 Converts Dorsal Midbrain Astrocytes into Functional Neurons In Vivo. J. Neurosci..

[247] Rivetti di Val Cervo P., Romanov R.A., Spigolon G., Masini D., Martin-Montanez E., Toledo E.M., La Manno G., Feyder M., Pifl C., Ng Y.H. (2017). Induction of functional dopamine neurons from human astrocytes in vitro and mouse astrocytes in a Parkinson’s disease model. Nat. Biotechnol..

[248] Jorstad N.L., Wilken M.S., Grimes W.N., Wohl S.G., VandenBosch L.S., Yoshimatsu T., Wong R.O., Rieke F., Reh T.A. (2017). Stimulation of functional neuronal regeneration from Muller glia in adult mice. Nature.

[249] Yao K., Qiu S., Wang Y.V., Park S.J.H., Mohns E.J., Mehta B., Liu X., Chang B., Zenisek D., Crair M.C. (2018). Restoration of vision after de novo genesis of rod photoreceptors in mammalian retinas. Nature.

[250] An H., Lee H.-L., Cho D.-W., Hong J., Lee H.Y., Lee J.M., Woo J., Lee J., Park M., Yang Y.-S. (2020). TRANsCre-DIONE transdifferentiates scar-forming reactive astrocytes into functional motor neurons. bioRxiv.

[251] Qian H., Kang X., Hu J., Zhang D., Liang Z., Meng F., Zhang X., Xue Y., Maimon R., Dowdy S.F. (2020). Reversing a model of Parkinson’s disease with in situ converted nigral neurons. Nature.

[252] Zhou H., Su J., Hu X., Zhou C., Li H., Chen Z., Xiao Q., Wang B., Wu W., Sun Y. (2020). Glia-to-Neuron Conversion by CRISPR-CasRx Alleviates Symptoms of Neurological Disease in Mice. Cell.

